# Tumor-targeted delivery of a STING agonist improves cancer immunotherapy

**DOI:** 10.1073/pnas.2214278119

**Published:** 2022-11-29

**Authors:** You-tong Wu, Yan Fang, Qi Wei, Heping Shi, Huiling Tan, Yafang Deng, Zhiqun Zeng, Jian Qiu, Chuo Chen, Lijun Sun, Zhijian J. Chen

**Affiliations:** ^a^ImmuneSensor Therapeutics, Dallas, TX 75235; ^b^Department of Molecular Biology, University of Texas Southwestern Medical Center, Dallas, TX 75390; ^c^Center for Inflammation Research, University of Texas Southwestern Medical Center, Dallas, TX 75390; ^d^Department of Biochemistry, University of Texas Southwestern Medical Center, Dallas, TX 75390; ^e^HHMI, Chevy Chase, MD 20815

**Keywords:** cGAS, STING, ADC, cancer, tumor immunity

## Abstract

The cGAS-STING pathway is essential for immune defense against microbial pathogens and malignant cells; as such, STING is an attractive target for cancer immunotherapy. However, systemic administration of STING agonists poses safety issues while intratumoral injection is limited by tumor accessibility. Here, we generated antibody-drug conjugates (ADCs) by conjugating a STING agonist through a cleavable linker to antibodies targeting tumor cells. Systemic administration of these ADCs was well tolerated and exhibited potent antitumor efficacy in syngeneic mouse tumor models. The STING ADC further synergized with an anti-PD-L1 antibody to achieve superior antitumor efficacy. The STING ADC promoted multiple aspects of innate and adaptive antitumor immune responses, including activation of dendritic cells, T cells, natural killer cells and natural killer T cells, as well as promotion of M2 to M1 polarization of tumor-associated macrophages. These results provided the proof of concept for clinical development of the STING ADCs.

## Significance

The cGAS-STING pathway plays a key role in antitumor immunity. Several STING agonists have shown antitumor efficacy in preclinical studies and are under clinical development. However, systemic administration of STING agonists may have adverse effects by inducing excessive cytokines. In this study, a STING agonist was conjugated to an antibody against a tumor-associated antigen, EGFR, to generate antibody-drug conjugate (ADC). Systemic administration of the STING ADC in mouse tumor models was well tolerated and exhibited potent antitumor efficacy, which was further enhanced when combined with an anti-PD-L1 antibody. Mechanistic studies revealed that the STING ADC activated multiple aspects of antitumor immune responses. This study sets the stage for clinical development of the STING ADCs.

The evolution of malignant cells under immune surveillance gives rise to tumors capable of escaping immune detection or suppression of immune attack (1). The core strategy in cancer immunotherapy is to overcome immune suppression and reinvigorate immune responses to cancer cells. One effective strategy to overcome immune suppression is the development of immune checkpoint inhibitors (2), including antibodies against cytotoxic T lymphocyte antigen 4, cluster of differentiation 47, programmed cell death 1, and programmed cell death 1 ligand 1 (PD-L1). Several of these antibodies have been approved by FDA for the treatment of various cancer types and have shown unprecedented efficacy and long-lasting response. However, immune checkpoint inhibitors are effective for only a small fraction (on average 20 to 30%) of cancer patients (2), suggesting that lifting negative signals alone is not sufficient in patients who do not have adequate antitumor immunity.

The role of cyclic GMP-AMP synthase (cGAS)-stimulator of interferon genes (STING) signaling pathway in cancer immunity has been increasingly recognized in recent years (3, 4). cGAS is an enzyme that can be activated by DNA from invading pathogens to synthesize 2′-3′-cyclic-GMP-AMP (cGAMP) from GTP and ATP (5). As a second messenger, cGAMP binds to STING (6, 7), which activates TANK-binding kinase 1 (TBK1) and inhibitor of κB kinases (IKKs). TBK1 and IKK in turn activate the transcription factors interferon regulatory factor 3 (IRF3) and nuclear factor κB (NF-κB), respectively. IRF3 and NF-κB then induce type I interferons (IFNs) and other inflammatory cytokines. STING also triggers autophagy through a mechanism independent of TBK1 and IKK (8). Although initially discovered as a pattern recognition receptor for DNA from invading microbes, cGAS can also be activated by self-DNA under some conditions, leading to autoimmune disorders (9–11). In tumors, DNA from dying cancer cells can gain access to the cytosol of antigen-presenting cells and activate the cGAS-STING pathway, leading to induction of type I interferons and other immune stimulatory molecules, which promote antitumor immunity through activation of T cells and natural killer (NK) cells (12, 13). Interestingly, conventional cancer treatments including radiation (14) and certain chemotherapy (15), which cause various forms of DNA damage and genomic instability, also activate the cGAS pathway and generate antitumor-immune responses. In light of the importance of the cGAS-STING pathway in cancer immunity, considerable efforts have been undertaken to target this pathway pharmaceutically. As an endogenous and high-affinity ligand of STING, cGAMP itself has been shown to exhibit strong antitumor effects in syngeneic mouse tumor models (12, 16). Several cyclic dinucleotides (CDNs) derived from cGAMP, as well as non-nucleoside small molecule STING agonists, are at different stages of clinical development (17). However, a major concern in the clinical usage of STING agonists is the potential adverse effect associated with cytokine induction, particularly when they are administered systemically, which requires a higher dose in order to reach an effective concentration in tumors. This largely constrains the administration routes to intratumoral injection, which has limitations including the accessibility of tumors and insufficient retention time in tumors. Systemic delivery of STING agonists that are targeted to tumors may overcome such limitations.

Antibody-drug conjugates (ADCs) are a category of drugs in which a therapeutic compound (payload) is conjugated to a monoclonal antibody through a suitable linker (18). ADCs take the advantage of the specificity of an antibody, which usually recognizes a cell surface molecule over-expressed in malignant cells, to deliver payloads to tumors. This strategy concentrates the payloads in tumors and reduces unwanted exposure to normal tissues. Currently, 14 ADCs have been approved for clinical use, and many more are under clinical development (19). The majority of ADCs currently under development carry cytotoxic payloads that directly kill cancer cells. In this study, we designed and synthesized a STING agonist that is conjugated through a cleavable linker to antibodies targeting epidermal growth factor receptor (EGFR), which is overexpressed in a wide range of cancer cells (20). The resulting ADC activated immune cells in vitro with high potency and showed strong therapeutic efficacy with minimal toxicity in syngeneic mouse tumor models. Our study unveiled a category of ADCs that has promising clinical potential.

## Results

### STING ADCs Stimulate Interferon Response in Cells Expressing EGFR.

**Fig. 1.**
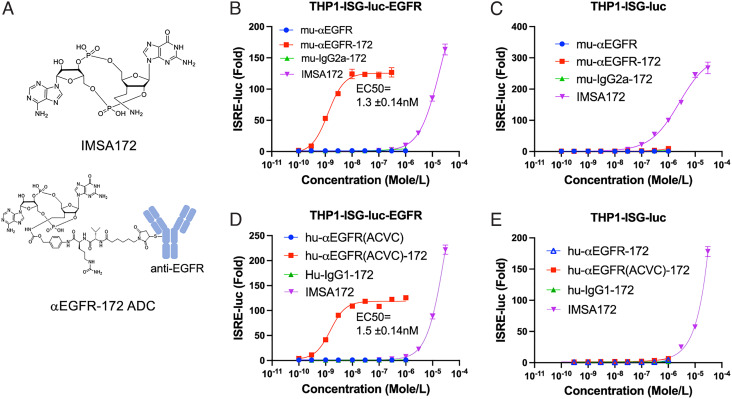
Chemical structures and immunostimulatory activities of IMSA172 and its ADCs in cells. (*A*) Diagrams of chemical structures of IMSA172 and anti-EGFR-172 ADC used in this study. (*B*–*E*) Serial dilutions of IMSA172 or its conjugates with the indicated antibodies (ADCs) were incubated with THP1-ISG-Luc (*C* and *E*) or this cell line stably expressing human EGFR (THP1-ISG-luc-EGFR; *B* and *D*) for 16 h, and the interferon response was measured by luciferase assay (see *Materials and Methods*). mu-αEGFR-172: mouse anti-human EGFR conjugated to IMSA172; mu-IgG2a-172: mouse IgG2a conjugated to IMSA172; mu-αEGFR: unconjugated mouse anti-human EGFR; Hu-αEGFR: human anti-human EGFR (same as cetuximab); hu-αEGFR-172: human anti-human EGFR antibody conjugated to IMSA172; hu-IgG-172: human IgG1 conjugated to IMSA172; hu-αEGFR(ACVC): hu-αEGFR-172 with A114C and V205C mutations for site-specific conjugation; hu-αEGFR(ACVC)-172: hu-αEGFR(ACVC) conjugated to IMSA172 via four specific cysteines. EC50 values were derived using the curve fitting function in Graphpad. Data are representative of at least three independent experiments.

To overcome the adverse effects associated with systemic administration of STING agonists, we sought targeted delivery of the payload to tumor sites by conjugation to an antibody against EGFR, which is overexpressed in multiple tumor types (20). We synthesized the cGAMP analogue IMSA172, which contains an aminoethyl group at the 3’-position of guanosine instead of a hydroxyl group as in cGAMP (Fig. 1*A* and *SI Appendix*, Figs. S1 and S2). The amino group allows for conjugation of IMSA172 to an antibody through a self-immolative linker that releases the payload in a traceless manner after the ADC is taken up by cells (*SI Appendix*, Fig. S3) (21). The activity of IMSA172 was assayed in a reporter cell line designated THP1-ISG-luc, which is a human monocytic cell line THP1 harboring a luciferase reporter gene under the control of a promoter comprised of five IFN-stimulated response elements (ISRE) fused to an ISG54 minimal promoter. In this cell line, IMSA172 induced IFN response with a half-maximal effective concentration (EC_50_) of 35 μM, similar to the potency of cGAMP (EC_50_= 23 μM) (*SI Appendix*, Fig. S4*A*). In the presence of perfringolysin O (PFO), a pore-forming toxin allows free entry of small molecules through the plasma membrane (5, 22), IMSA172 displayed an EC_50_ of ~11 nM, similar to that of cGAMP (EC_50_= 9.6 nM; *SI Appendix*, Fig. S4*B*). To prepare STING ADCs, IMSA172 was conjugated to EGFR antibodies through a cleavable maleimidocaproyl-valyl-citrullinyl-p-aminobenzyloxycarbonyl (Mc-Val-Cit-PABC) linker, which reacts with the amino group of IMSA172 on one side, and the sulfhydryl group of cysteine residues of the antibodies on the other side (Fig. 1*A* and *SI Appendix*, Fig. S3). Several versions of EGFR antibodies and isotype controls were chosen to make STING ADCs. IMSA172 was conjugated to a murine-anti-human EGFR antibody (mu-αEGFR) and its isotype control mouse IgG2a, which generated the ADCs named mu-αEGFR-172 and mu-IgG2a-172, respectively, both with a drug-to-antibody ratio (DAR) of approximately 3. The same strategy was used to conjugate IMSA172 to a human-anti-human EGFR antibody (designated as hu-αEGFR, identical to cetuximab (23)) and its isotype control human-IgG1, which generated ADCs designated as hu-αEGFR-172 and hu-IgG1-172, respectively, both with a DAR around 4. A mutant version of cetuximab harboring an alanine to cysteine mutation at amino acid 114 (A114C) on heavy chains and a valine to cysteine mutation at amino acid 205 (V205C) on light chains, designated as hu-αEGFR(ACVC), provides four cysteines on each antibody and was used for site-specific conjugation (see *Materials and Methods*) (24); the resulting ADC is designated as hu-αEGFR(ACVC)-172, with a DAR of 4. Immunostimulatory activity of these ADCs was tested in THP1-ISG-luc cells stably expressing human EGFR, designated as THP1-ISG-luc-EGFR. In this cell line, all three ADCs targeting EGFR induced robust interferon responses, with EC_50_ in the range of 1–2 nM, which was more than 10,000-fold lower than the free payload, IMSA172 (Fig. 1 *B* and *D* and *SI Appendix*, Fig. S4*C*). Unconjugated EGFR antibodies did not induce any response, indicating the stimulation depends on IMSA172 (Fig. 1 *B* and *D* and *SI Appendix*, Fig. S4*C*). EGFR ADCs were unable to induce an interferon response in THP1-ISG-luc lacking EGFR expression (Fig. 1 *C* and *E*), indicating that the ADC activity was dependent on EGFR expression on the target cells. In support of this, isotype control ADCs from either murine or human IgG, mu-IgG2a-172 and h-IgG1-172, did not induce an interferon response in THP1-ISG-EGFR cells (Fig. 1 *B* and *D*). Cellular activity of the EGFR-172 ADC was inhibited by competition with an unconjugated EGFR antibody but not a control IgG1 (*SI Appendix*, Fig. S4*D*), further underscoring the target specificity of these ADCs. In a murine RAW 264.7 macrophage cell line harboring a luciferase reporter gene under the control of the ISRE promotor (Raw-ISG-luc), both human and murine EGFR-172 ADCs induced IFN response with EC_50_ values of ~0.5 μM, which were about ten times lower than the payload IMSA172 (*SI Appendix*, Fig. S5 *A* and *B*). Because this cell line does not express human EGFR, the activation is likely mediated by FcγR binding and uptake of ADCs. Indeed, both mu-αEGFR-172 and the control ADC hu-IgG1-172 induced comparable responses, which were inhibited by antibodies against CD16 (FcγRIII) and CD32 (FcγRII) on the reporter cells (*SI Appendix*, Fig S5 *C* and *D*). Notably, the activity of EGFR-172 ADC in Raw-ISG-luc cells was >100 fold lower than its activity in THP1-ISG-luc-EGFR cells, suggesting that an ADC taken up by cells through a tumor antigen is more effective at activating the STING pathway than an ADC taken up through an Fc receptor.

### STING ADCs Exert Potent Antitumor Effects in Mouse Models.

**Fig. 2.**
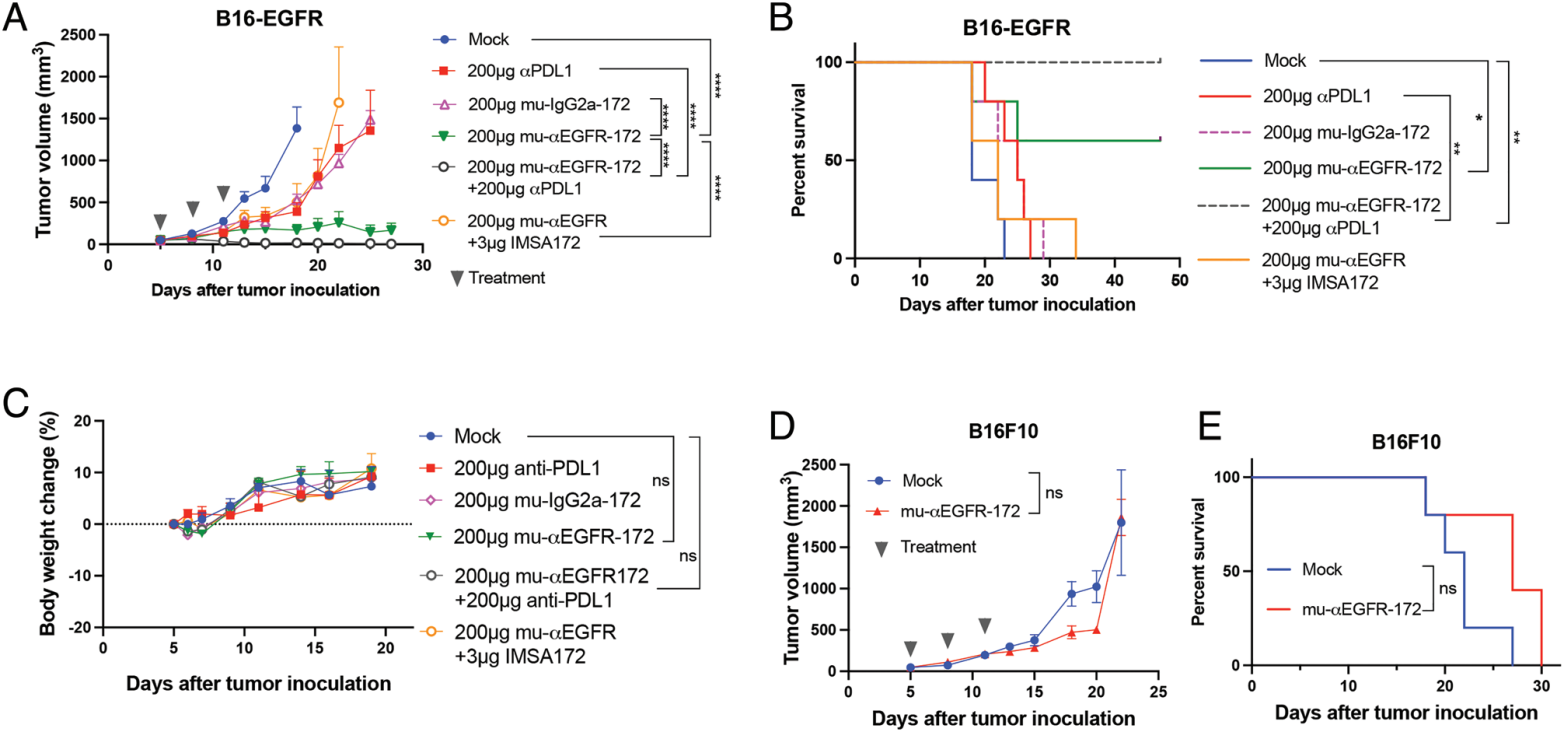
Antitumor efficacy of mu-αEGFR-172. (*A* and *B*) Groups of C57BL/6 mice (n = 5) with established B16F10 tumors expressing human EGFR were treated with the indicated ADCs or PD-L1 antibody or both via intraperitoneal injection. In one group, mice were treated with PBS as mock control. In another group, mu-αEGFR was mixed with IMSA-172 without conjugation. Tumor growth (*A*) and mouse survival (*B*) were monitored. (*C*) Body weight change (%) of mice after treatments. (*D* and *E*) Groups of C57BL/6 mice (n = 5) with B16F10 (not expressing EGFR) tumors were treated with mu-αEGFR-172 via intraperitoneal injection. Tumor growth (*D*) and mouse survival (*E*) were monitored. Tumor growth and body weights are represented as mean ± SEM, ns: not significant, *P < 0.05, **P < 0.01, ***P < 0.001, ****P < 0.0001 (two-way ANOVA). Log rank (Mantel-Cox) test was used for survival data, ns: not significant, *P < 0.0332, **P < 0.0021, ***P < 0.0002, ****P < 0.0001.

To test the effect of STING ADCs in vivo, we used a mouse tumor model in which the C57BL/6J mice were implanted subcutaneously with B16F10 melanoma cells stably expressing human EGFR (B16-EGFR). After the tumors grew to ~100 mm^3^, STING ADCs were administered to the mice through intraperitoneal injection three times (200 μg each time on day 5, 8, and 11 after tumor cell inoculation). The treatment with mu-αEGFR-172 alone significantly suppressed tumor growth (Fig. 2*A*) and led to complete tumor remission in 60% of mice (Fig. 2*B*). In contrast, treatment with an anti-PD-L1 antibody only modestly reduced tumor growth and slightly improved mouse survival in this model. Strikingly, the combination of the αEGFR-172 ADC with the anti-PD-L1 antibody completely suppressed tumor growth and led to the survival of all mice, suggesting a synergy between these two treatments (Fig. 2 *A* and *B*). Both the ADC alone and its combination with the anti-PD-L1 antibody were well tolerated in these mice, as indicated by the continued weight gains after multiple treatments (Fig. 2*C*).

Several control groups were included to evaluate the specificity of ADC treatment. Compared to the mu-αEGFR-172 ADC, a mixture of mu-αEGFR antibody and free payload IMSA172, which contained equal amounts of the unconjugated antibody and payload as in the ADC treatment group, had only a limited effect on tumor growth and marginal improvement of mouse survival when compared to the phosphate-buffered saline (PBS)-treated group (mock; Fig. 2 *A* and *B*). A control ADC generated from mouse IgG2a (mu-IgG2a-172) only partially inhibited tumor growth and slightly extended the mouse survival (Fig. 2 *A* and *B*). This partial antitumor effect is likely due to the FcγR-mediated uptake of the ADC by innate immune cells in tumors (*SI Appendix*, Fig. S5 *C* and *D*). The mu-αEGFR-172 ADC failed to inhibit tumor growth or improve mouse survival when the B16F10 cell line did not express human EGFR (Fig. 2 *D* and *E*). These results demonstrate that the antitumor efficacy of the αEGFR-172 ADC was dependent on the specificity of the EGFR antibody and its conjugation with IMSA172.

**Fig. 3.**
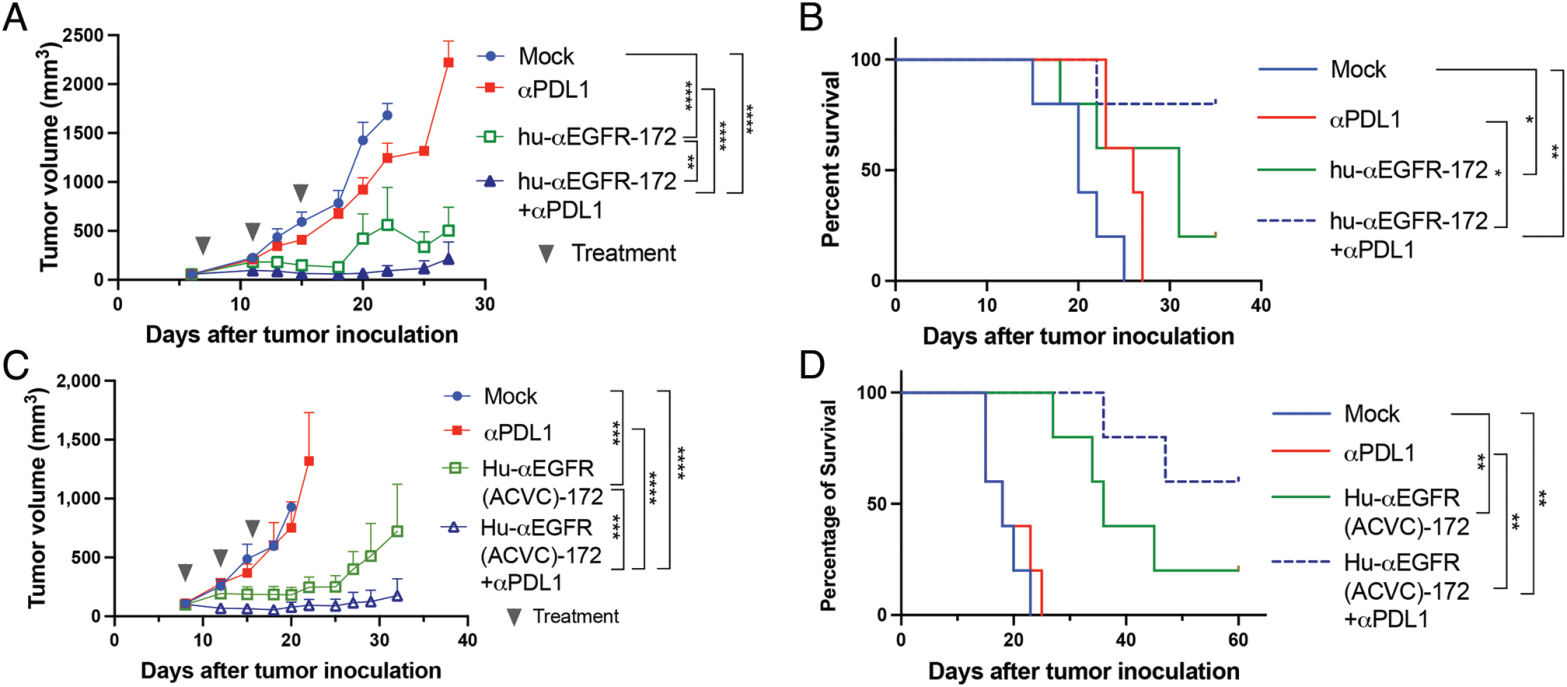
Antitumor efficacy of human anti-EGFR-172 ADCs. (*A* and *B*) Groups of C57BL/6 mice (n = 5) with established B16F10-EGFR tumors were treated intraperitoneally with hu-αEGFR-172 or anti-PD-L1 antibody or both as indicated; tumor growth (*A*) and mouse survival (*B*) were monitored. (*C* and *D*) Similar to (*A*) and (*B*) except that the hu-αEGFR(ACVC)-172 ADC was used. Tumor growth data are presented as mean ± SEM, ns: not significant, *P < 0.05, **P < 0.01, ***P < 0.001, ****P < 0.0001 (two-way ANOVA). Log rank (Mantel-Cox) test was used for survival data, ns: not significant, *P < 0.0332, **P < 0.0021, ***P < 0.0002, ****P < 0.0001.

Moving toward clinical development, we generated STING ADCs using the FDA-approved human EGFR antibody, cetuximab. Both hu-αEGFR-172 and hu-EGFR(ACVC)-172 markedly inhibited tumor growth (Fig. 3 *A* and *C*) and extended the survival of mice bearing the B16F10-EGFR tumors (Fig. 3 *B* and *D*). The efficacy of both ADCs was further enhanced by combination with the PD-L1 antibody, which led to complete tumor remission in 4/5 and 3/5 of the mice when combined with hu-αEGFR-172 and hu-αEGFR(ACVC)-172, respectively. The hu-αEGFR(ACVC)-172 ADC was also tested in another syngeneic tumor model using the MC38 colon cancer cell line stably expressing EGFR (*SI Appendix*, Fig. S6). In this model, the ADC alone effectively controlled tumor growth and led to tumor-free survival of 60% of mice.

### Pharmacokinetic (PK) and Pharmacodynamic (PD) Properties of STING ADC.

**Fig. 4.**
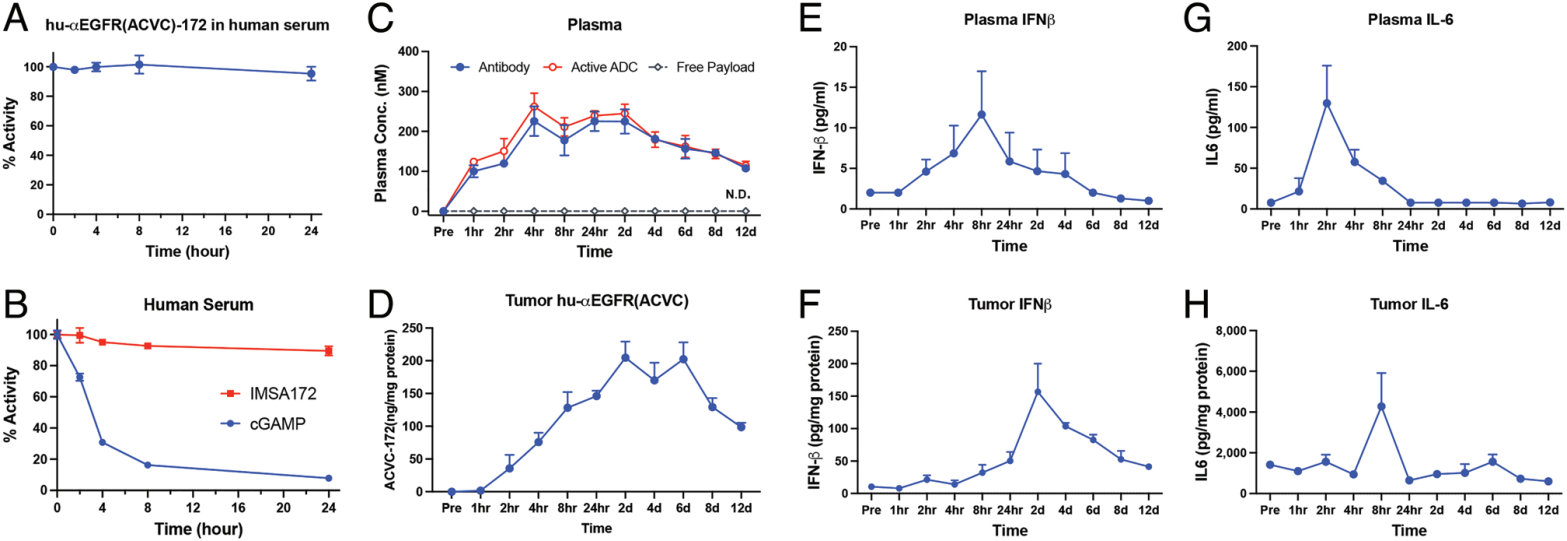
PK and PD of hu-αEGFR(ACVC)-172. (*A*) The stability of hu-αEGFR(ACVC)-172 in human sera. hu-αEGFR(ACVC)-172 was incubated with human sera over a time course as indicated. The remaining activity was measured in THP1-ISG-luc-EGFR reporter cells and presented as percentage of untreated samples. (*B*) Similar to (*A*) except that the payload IMSA172 and cGAMP were tested in the human sera. (*C*–*H*) PK and PD of hu-αEGFR(ACVC)-172 in mice. Mice-bearing B16-EGFR tumors were injected with 100 μg of hu-αEGFR(ACVC)-172 intraperitoneally and three mice were sacrificed at each timepoint to collect blood and tumors. ADC and cytokine levels were measured from plasma and tumor homogenates using ELISA. The activity of the ADC and free payload (IMSA172) was measured in THP1-ISG-luc-EGFR cells. Data are presented as mean ± SEM. N.D.: non-detectable.

The stability of αEGFR(ACVC)-172 ADC was evaluated in human sera. Incubation of this ADC with human sera for up to 24 h did not reduce its activity (Fig. 4*A*). The payload IMSA172 also showed good stability in the same serum for 24 h while cGAMP rapidly lost its activity (Fig. 4*B*), consistent with previous reports showing that cGAMP is degraded in the sera by ENPP1 (25). For in vivo PK/PD studies, mice were injected with 100 μg of hu-αEGFR(ACVC)-172 intraperitoneally, followed by the collection of plasma and tumor samples at different time points. Total hu-αEGFR(ACVC) antibody and active ADC in the plasma, as quantified, respectively, by ELISA and a bioassay for STING activation in THP1-ISG-luc-EGFR cells (see *Materials and Methods*), reached peak levels 4 h after injection, maintained at high levels for 2 d, and slowly decreased in the following days. On day 12, the concentration was still about half of the peak level (Fig. 4*C*). This PK profile of the ADC was similar to that of unconjugated EGFR antibody and is typical of other antibodies (26). No release of the free payload in the sera was detected (Fig. 4*C*). In tumors, a steady increase of the antibodies in tumors was observed up to day 6 (Fig. 4*D*), followed by a slow reduction over time, indicating the drug was indeed targeted to tumor sites. We also measured the production of cytokines including interferon-β (IFNβ) and interleukin-6 (IL-6) in the plasma and tumor homogenates as PD markers. In plasma, low levels of IFNβ were detected which peaked at 8 h after dosing and decreased at subsequent time points. For IL-6, there was a transient and modest increase 2–4 h after dosing, followed by a rapid decrease to undetectable levels in 24 h (Fig. 4 *E* and *G*). In tumors, IFNβ levels (Fig. 4*F*) peaked at 2 d after treatment and slowly decreased in the following days; this profile paralleled that of ADC in the tumors (Fig. 4*D*). The tumor IL-6 levels (Fig. 4*H*) peaked at 8 h after treatment and rapidly decreased to baseline in subsequent time points.

### STING ADC Activates T Cells, NK Cells, and NKT Cells in Tumors.

To investigate the mechanisms by which STING ADC promotes antitumor immunity, we established B16-EGFR tumors in mice and treated the mice with the hu-αEGFR(ACVC)-172 ADC or PBS as control. Two days after one treatment or 6 d after two treatments with 3 d interval, tumors and draining lymph nodes (dLNs) were collected to harvest cells for fluorescence-activated cell sorting (FACS) analysis of immune cell populations and their activation markers. The FACS gating strategies are shown in *SI Appendix*, Fig. S7.

**Fig. 5.**
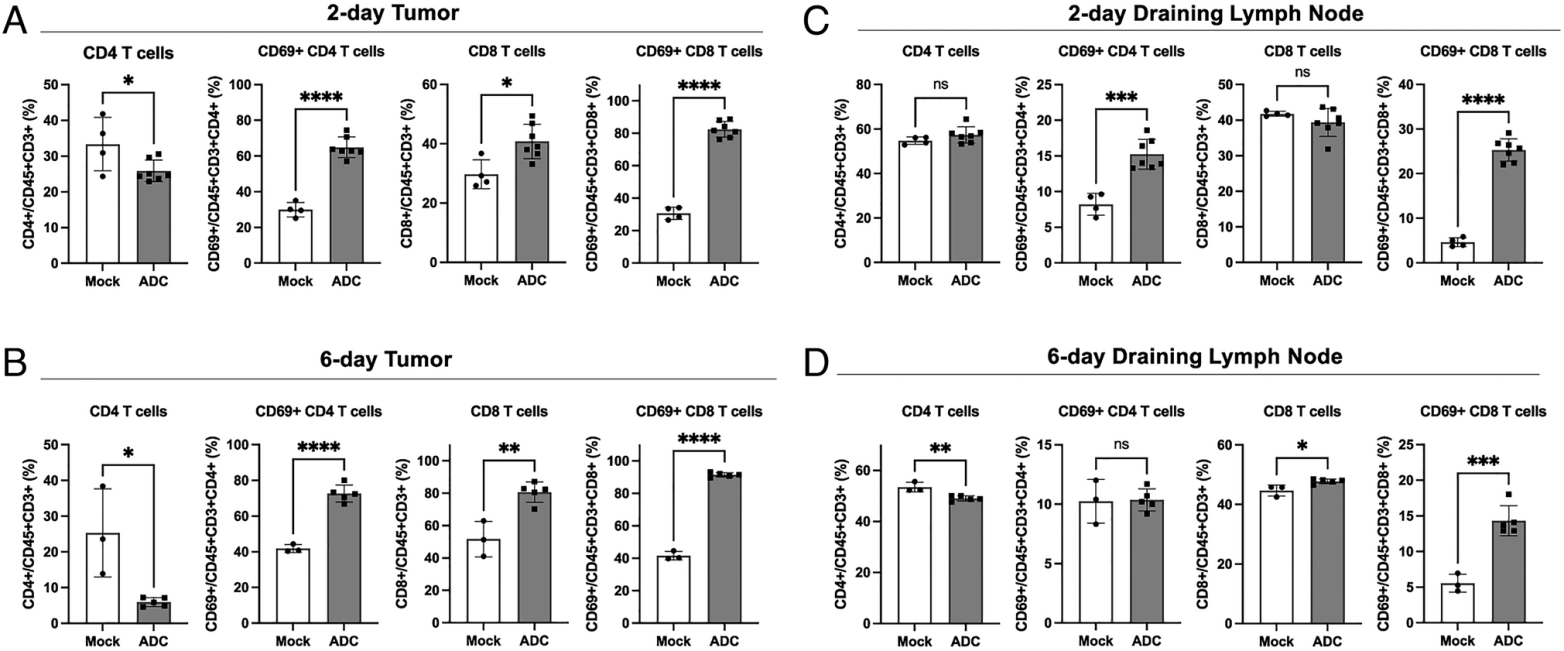
ADC treatment activated T cells in tumors and draining lymph nodes and increased CD8/CD4 ratio in tumors. B16F10-EGFR cells were inoculated into C57BL/6 mice subcutaneously. (*A* and *C*) On day 7, 200 μg of hu-αEGFR(ACVC)-172 (ADC) or PBS (mock) was administrated intraperitoneally. Two days later, cells from tumors (*A*) and draining lymph nodes (*C*) were analyzed by flow cytometry using antibodies against the indicated T cell markers. Mock: n = 4, ADC: n = 7. (*B* and *D*) The tumor-bearing mice were treated with ADC or mock on day 7 and day 10. Six days after the first treatment, cells from tumors (*B*) and draining lymph nodes (*D*) were analyzed by flow cytometry. Mock: n = 3, ADC: n = 5. Data are shown as mean ± SD and individual values. ns: not significant, *P < 0.05, **P < 0.01, ***P < 0.001, ****P < 0.0001 (student’s *t  *test).

While no significant differences were observed in the numbers of tumor-infiltrating leukocytes (CD45+) and T cells (CD3+) between mock and STING ADC-treated mice (*SI Appendix*, Fig. S8 *A* and *B*), there was a significant decrease of CD4 T cells and an increase of CD8 T cells in their percentages of total T cells (CD3+) in tumors in response to the ADC treatment (Fig. 5 *A* and *B* and *SI Appendix*, Fig. S8*C*). Moreover, there were marked increases (>two-fold) of activated CD4 and CD8 T cell populations in the tumors both 2 d and 6 d after the ADC treatment. Similarly, the populations of activated CD4 and CD8 T cells increased in the dLN 2 d after the ADC treatment (Fig. 5*C* and *SI Appendix*, Fig. S8 *D* and *E*). Activated CD8 but not CD4 T cell populations increased in the dLN 6 d after the ADC treatment (Fig. 5*D* and *SI Appendix*, Fig. S8 *D* and *E*). These results show that the STING ADC activated both CD4 and CD8 T cells and increased the percentage of CD8 T cells but decreased that of CD4 T cells in the tumors.

**Fig. 6.**
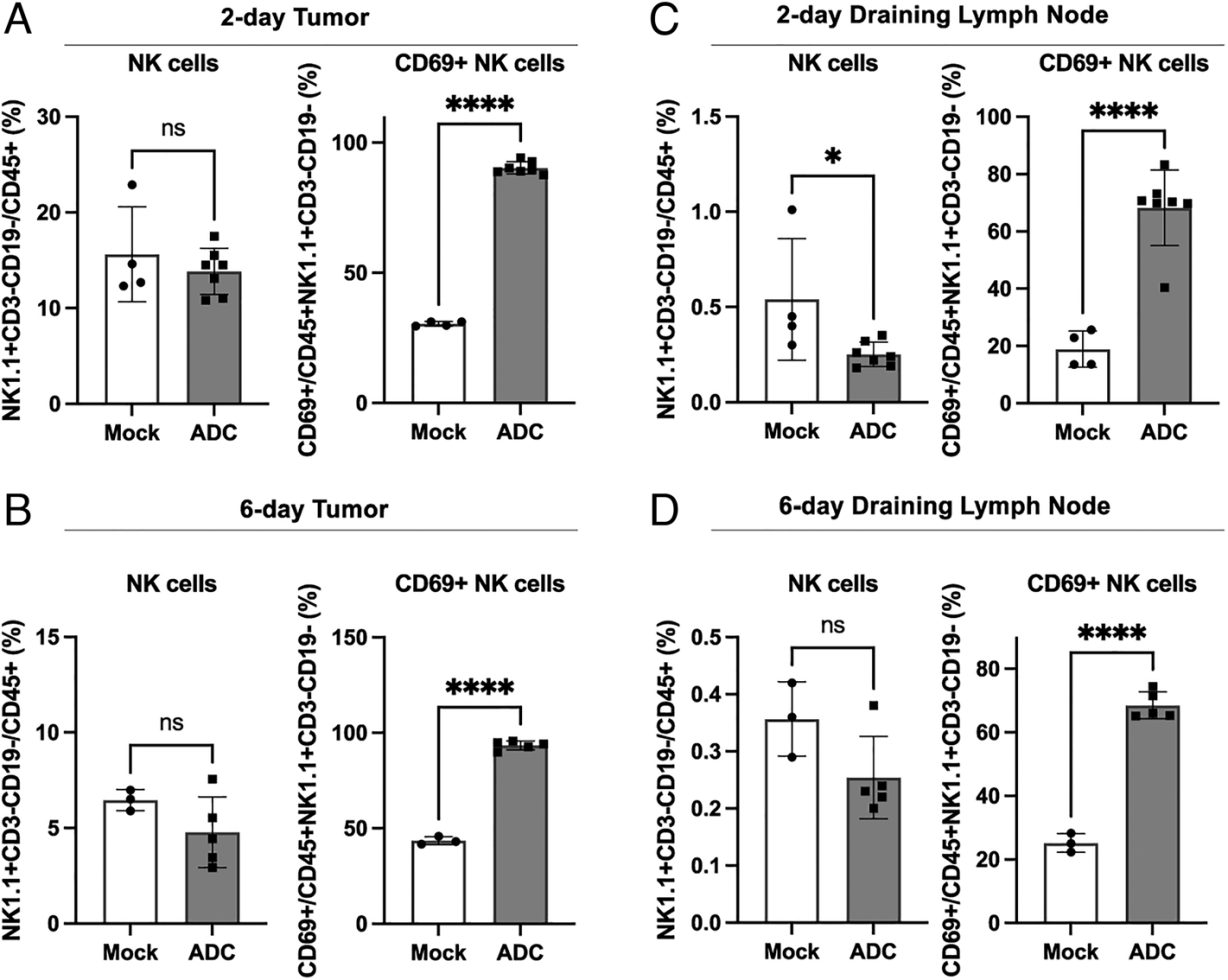
ADC treatment activated NKcells in both tumors and draining lymph nodes. Experiments were performed as described in Fig. 5, except that the flow cytometry analysis was focused on NK cells (NK1.1+CD3−CD19−) 2 days (*A*) or 6 days (*B*) in tumor and 2 days (*C*) and 6 days (*D*) in draining lymph nodes after ADC treatments. ns: not significant, *P < 0.05, ****P < 0.0001 (student’s *t *test).

Analyses of NK cells (NK1.1+CD3−) showed that although the STING ADC treatment did not change the number of these cells in the tumors or dLN, there was a marked increase (>two-fold) in the percentage of activated (CD69+) NK cells in response to the ADC treatment (Fig. 6 and *SI Appendix*, Fig. S9). Similarly, the percentage of activated NKT cells (NK1.1+CD3+) in the tumors and dLN increased significantly (>two-fold) both 2 d and 6 d after the ADC treatment (*SI Appendix*, Fig. S10). Thus, the STING ADC activated both NK and NKT cells, which likely contributed to the antitumor effects.

### STING ADC Activates Dendritic Cells (DCs) and Promotes Macrophage Polarization.

**Fig. 7.**
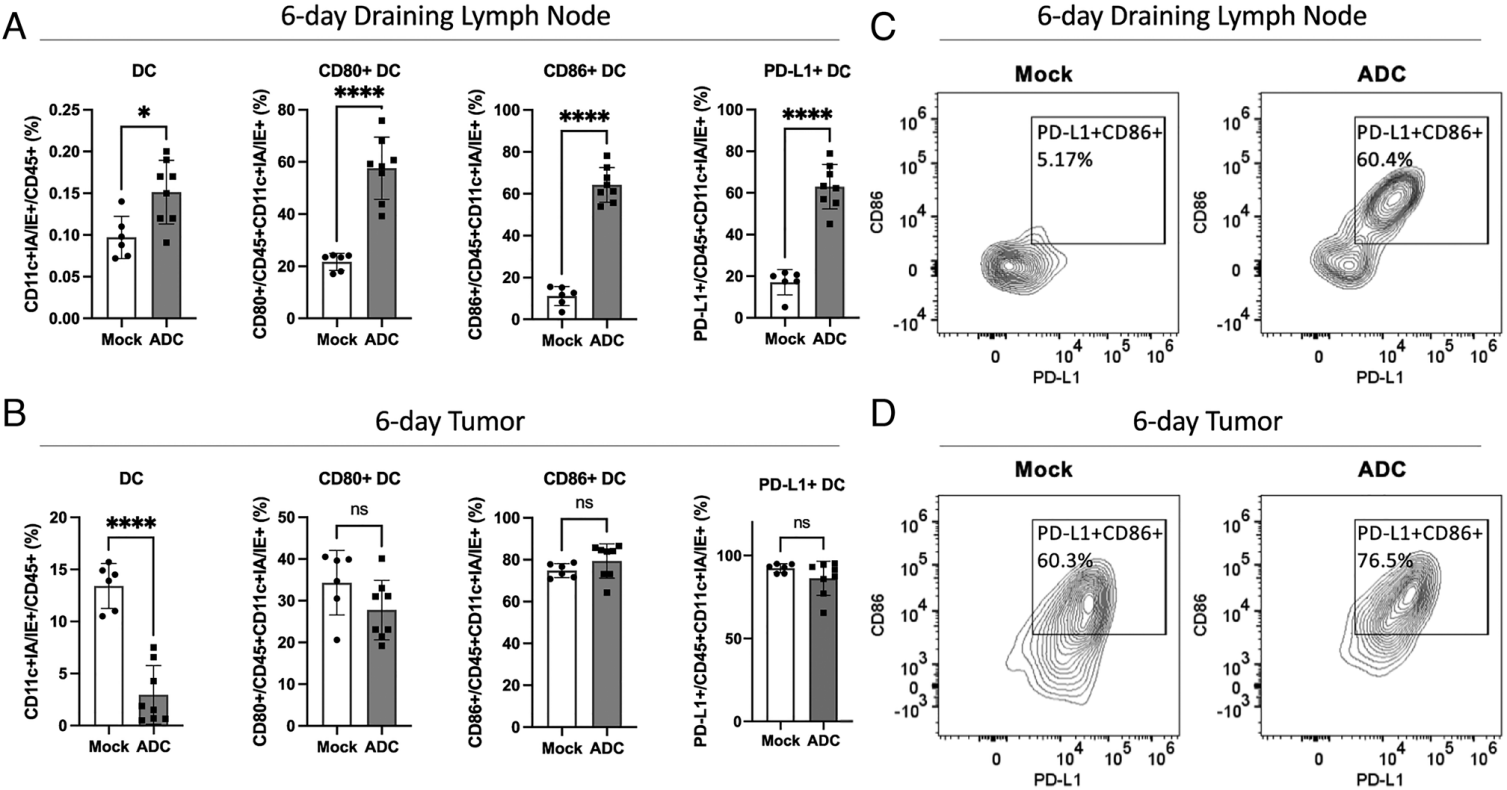
ADC treatment activated DCs and enhanced their migration from tumors to draining lymph nodes. Experiments were performed similarly to Fig. 5 *B* and *D*. Six days after the first treatment, cells from draining lymph nodes (*A*) and tumors (*B*) were analyzed by flow cytometry focusing on DCs (CD11c+IA/IE+). Data are shown as the mean ± SD and individual values. Mock: n = 6, ADC: n = 8. ns: not significant, *P < 0.05, ****P < 0.0001 (student’s *t *test). (*C* and *D*) Representative FACS plots of CD86 and PD-L1 expression levels on DCs from draining lymph nodes (*C*) and tumors (*D*).

**Fig. 8.**
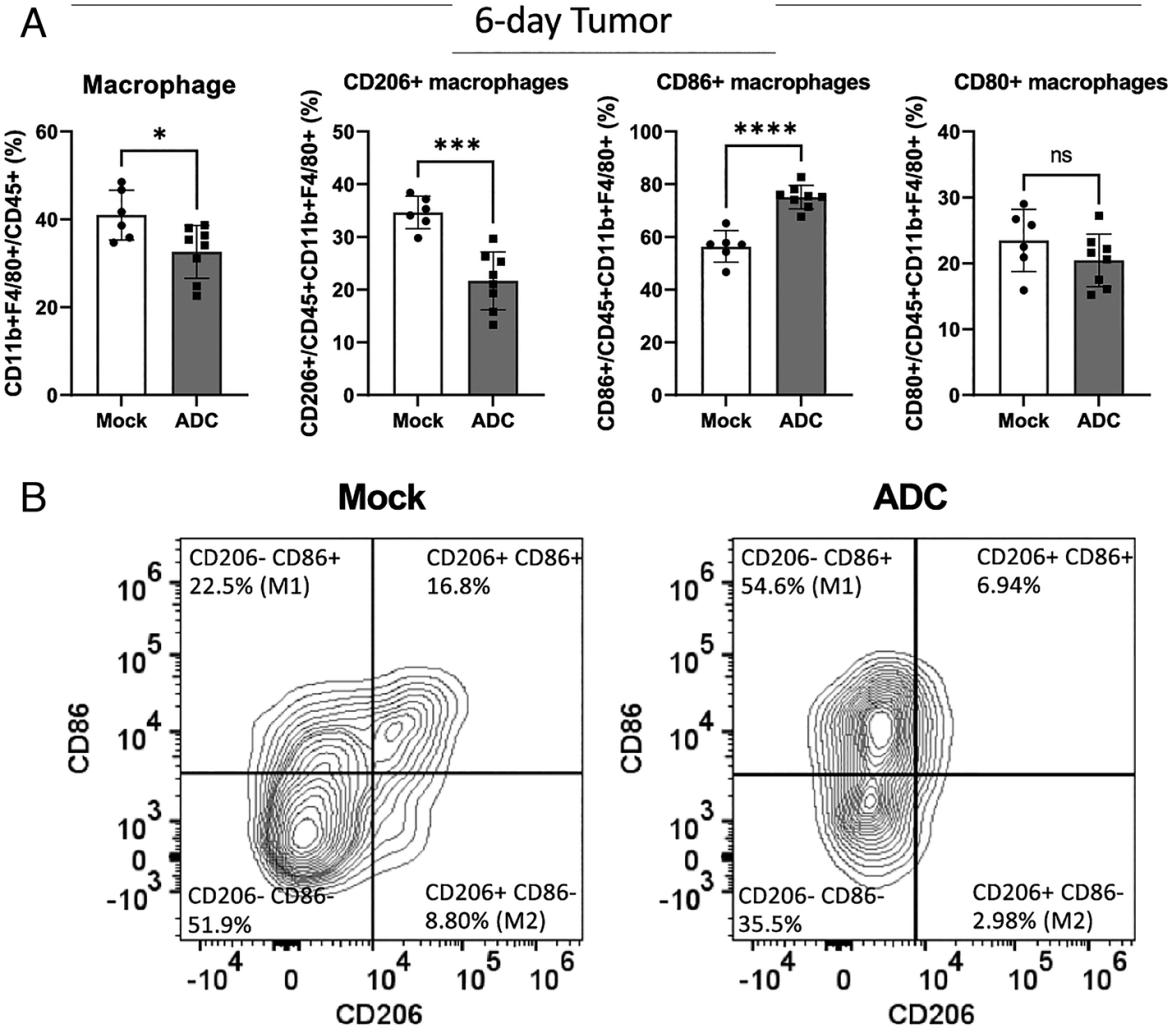
ADC treatment polarized M2-like macrophages to M1-like macrophages in tumors. (A) Experiments were performed as described in Fig. 7 except that the flow cytometry analysis was focused on tumor-associated macrophages (CD11b+F4/80+). ns: not significant, *P < 0.05, ***P < 0.001, ****P < 0.0001 (student’s *t *test). (*B*) Representative FACS plots of CD86 and CD206 expression levels in tumor-associated macrophages.

To further investigate how STING ADCs activate antitumor immune responses, we analyzed DCs (CD11c+IA/IE+) and macrophages (CD11b+F4/80+) in the tumors and dLN. Two days after the ADC treatment, there was a marked increase in the surface expression of the co-stimulatory molecules CD80 and CD86 as well as PD-L1 on the DCs in the tumors and dLN (*SI Appendix*, Fig. S11), indicating DC activation. Moreover, the total DC population decreased in the tumors (Fig. 7 *B*, *Left* panel) and increased in the dLN (Fig. 7 *A*, *Left* panel) 6 d after the ADC treatment, suggesting a migration of DCs from the tumors to the dLN, where they might activate T cells. At this time, the expression of CD80, CD86, and PD-L1 were highly elevated in the dLN (Fig. 7*A*) but not in the tumors (Fig. 7*B*), consistent with the migration of activated DCs from the tumors to the dLN. The STING ADC-induced upregulation of PD-L1 on the DCs provides a mechanistic basis for the synergistic antitumor effect of combining the STING ADC and PD-L1 antibodies (Fig. 8).

Tumor-associated macrophages (TAM) play a key role in modulating the tumor microenvironment. TAM can polarize into two subtypes with opposite functionality (27). M1 macrophages, which are characterized by elevated surface expression of CD80 or CD86 and reduced expression of CD206, exert antitumor effects through their cytotoxic activities and antigen presentation. In contrast, M2 macrophages, which have elevated expression of CD206 and reduced expression of CD80 and CD86, promote tissue repair and tumor cell proliferation and suppress antitumor immunity. We found that 2 d after the ADC treatment, there was an increase of macrophages in the tumors as well as elevated expression of CD80 and CD86 in these macrophages, but the CD206 levels remained largely unchanged (*SI Appendix*, Fig. S12*A*). Six days after the ADC treatment, there was an elevated expression of CD86 and reduced expression of CD206 in the tumor-associated macrophages. Similar macrophage polarization was also observed in the dLNs 2 d and 6 d after the ADC treatment (*SI Appendix*, Fig. S12 *B* and *C*). These results demonstrate that the STING ADC induced the M2 to M1 polarization of macrophages in the tumors as well as in the dLN, which provides another mechanism underlying the antitumor effect of the STING ADC.

## Discussion

As a major innate immunity pathway that detects cancer cells and activates antitumor immune responses, the cGAS-STING pathway has been an attractive target for developing therapeutics to improve the outcome of cancer treatments. Although STING agonists have been shown to exert potent effects in several mouse tumor models, their use in human clinical trials is limited to intratumor injection because of concerns of immunotoxicity that may be caused by systemic administration, especially at a higher dose. To overcome this limitation, we developed a STING agonist, IMSA172, which can be conjugated to a tumor-targeting agent such as an antibody against EGFR. We showed that the αEGFR-172 ADC potently activated the STING pathway in cells overexpressing EGFR. Moreover, systemic administration of the αEGFR-172 ADC in mice strongly inhibited the growth of B16F10 tumors in a manner that depends on the expression of EGFR in the tumor cells. The antitumor effect of the αEGFR-172 ADC was most pronounced when it was combined with an anti-PD-L1 antibody, which by itself was ineffective against the B16F10 tumors. The systemic administration of αEGFR-172 ADC appeared to be well tolerated in mice, but extensive toxicology studies in animals are required before the ADC can be moved forward for clinical evaluation.

STING activation leads to the induction of type-I interferons and co-stimulatory molecules such as CD80 and CD86, which are important for the activation of adaptive immune responses. Consistent with STING activation, the αEGFR-172 ADC strongly induced the surface expression of CD80 and CD86 as well as PD-L1 in DCs in the tumors and dLNs. The increase of PD-L1 in DCs is likely a feedback mechanism that prevents overactivation of the immune system, and it also provides the molecular basis for combining STING activation and immune checkpoint blockade to achieve a synergistic antitumor immune response.

In response to the αEGFR-172 ADC treatment, the DC population decreased in the tumors and increased in the dLN, suggesting that the activated DCs might capture antigens in the tumors and migrate to dLN where they presented the tumor antigens to T cells (28). Indeed, we observed strong activation of both CD4 and CD8 T cells in the tumors and dLNs after the ADC treatment. Interestingly, while the ADC treatment increased the numbers of CD8 T cells in the tumors, it decreased the tumor CD4 T cell population. These CD4 T cells likely include regulatory T cells, which are known to inhibit antitumor-immune response (29). Future experiments should further analyze the tumor antigen specificity and functionality of the CD4 and CD8 T cells to determine if the STING ADC enhances the priming and/or recruitment of T cells in the tumors.

The αEGFR-172 ADC potently activated NK and NKT cells which are known to suppress tumor growth. In addition, the αEGFR-172 ADC treatment promoted the polarization of tumor-associated macrophages from M2 to M1. Because macrophages are the most abundant leukocytes in tumors, the significant polarization of macrophages from M2 to M1 likely plays an important role in the antitumor effects of the STING ADC. Further research should determine the relative contributions of different immune cells, including T cells, NK, NKT, DCs, and macrophages, to the antitumor effects of STING ADCs in different mouse tumor models.

Our results with the αEGFR-172 ADC provide a proof of concept for delivering a STING agonist specifically to tumors. Because IMSA172 is in principle suitable for conjugation with a variety of antibodies and other tumor-targeting agents, future research with different ADCs containing the IMSA172 payload should reveal the safety and efficacy of activating STING in different types of tumors through systemic delivery of STING ADCs. As STING activation not only enhances antitumor immunity but also upregulates immune inhibitory molecules such as PD-L1, the rational combination of a STING ADC with other therapeutics such as immune checkpoint inhibitors is likely to provide better outcomes in cancer treatments.

## Materials and Methods

### Synthesis of IMSA172.

The reaction scheme for the synthesis of IMSA172 is shown in *SI Appendix*, Fig. S1 and the NMR analysis results in *SI Appendix*, Fig. S2. Detailed synthetic procedures are described below.

#### Guanosine S2.

To a solution of sodium hydride (60%, 27.1 g, 0.68 mol, 4.0 equiv) in tetrahydrofuran (500 mL) at –20°C was added **S1** (30) (78 g, 0.17 mol, 1.0 equiv) in tetrahydrofuran (200 mL) over 5 min and then stirred at 25°C for 2 h before benzyl bromide (60 mL, 86.9 g, 0.51 mol, 3.0 equiv) was added. After stirring at 80°C for 14 h, the reaction was quenched with saturated ammonium chloride (20 mL) at 0°C, diluted with water (400 mL), extracted with methylene chloride (400 mL × 3), dried over sodium sulfate, filtered, concentrated, and purified by silica gel column chromatography (petroleum ether/ethyl acetate, 15/1 to 5/1) to give the corresponding benzyl ether (90 g, 97% yield) as a white solid. MS [M+Na]^+^ 573.1.

To a solution of the benzyl ether obtained above (90 g, 0.16 mol, 1.0 equiv) in methylene chloride (300 mL) was added dichloroacetic acid (30 mL, 21.1 g, 0.16 mol, 1.0 equiv). After stirring at 25°C for 3 h, saturated sodium bicarbonate (500 mL) was added at 0°C and the mixture was extracted with methylene chloride (100 mL × 3), dried over magnesium sulfate, filtered, concentrated, and purified by silica gel column chromatography (petroleum ether/ethyl acetate, 5/1 to 2/1) to give the corresponding primary alcohol (44 g, 87% yield) as a yellow oil.

To a solution of the alco 0.37 mmol, 1.0 equiv) in hol obtained above (62 g, 0.20 mol, 1.0 equiv) in methylene chloride (200 mL) was added benzoyl chloride (35 mL, 42.4 g, 0.30 mol, 1.5 equiv) and triethylamine (56 mL, 40.7 g, 0.40 mol, 2.0 equiv). After stirring at 25°C for 1 h, the mixture was concentrated and purified by silica gel column chromatography (petroleum ether/ethyl acetate, 15/1 to 10/1) to give the corresponding benzoate (80 g, 96% yield) as a light yellow oil. MS [M+Na]^+^ 435.1.

To a solution of the benzoate obtained above (20 g, 0.049 mol, 1.0 equiv) in water (6 mL) was added acetic acid (28 mL, 29.4 g, 0.5 mol, 10.1 equiv). After stirring at 100°C for 5 h, the reaction was quenched with saturated sodium bicarbonate (2 L), extracted with methylene chloride (400 mL × 3), dried over sodium sulfate, filtered, and concentrated to give the crude diol (70 g, 97% yield) as a light yellow oil. MS [M+Na]^+^ 395.1.

To a solution of the crude diol obtained above (70 g, 0.19 mol, 1.0 equiv) in pyridine (300 mL) was added acetic anhydride (70 mL, 76.8 g, 0.75 mol, 4.0 equiv). After stirring at 60°C for 4 h, the reaction was quenched with saturated sodium bicarbonate, extracted with methylene chloride (300 mL × 3), dried over sodium sulfate, filtered, concentrated, and purified by silica gel column chromatography (petroleum ether/ethyl acetate, 10/1 to 5/1) to give the corresponding diacetate (80 g, 93% yield) as a white solid. MS [M+Na]^+^ 479.1.

To a solution of *N*^2^-isobutyrylguanine (18.9 g, 0.085 mol, 1.3 equiv) in acetonitrile (300 mL) was added bis(trimethylsilyl)acetamide (85 mL, 69.5 g, 0.34 mol, 5.2 equiv). After stirring at 65°C for 30 min, the mixture was concentrated and redissolved in acetonitrile (600 mL) before the acetate obtained above (30 g, 0.066 mol, 1 equiv) in acetonitrile (150 mL) and trimethylsilyl trifluoromethanesulfonate (18 mL, 21.9 g, 0.099 mol, 1.5 equiv) was added at –15°C. After stirring at 65°C for 16 h, the reaction mixture was concentrated and purified by silica gel column chromatography (petroleum ether/ethyl acetate, 5/1 to 1/1) to give **S2** (30 g, 74% yield) as a white solid. ^1^H NMR (400MHz, CDCl_3_) δ 12.00 (s, 1H), 9.11 (s, 1H), 7.92–7.84 (m, 2H), 7.82–7.76 (m, 1H), 7.58 (t, *J* = 7.1 Hz, 1H), 7.46–7.37 (m, 2H), 7.27–7.16 (m, 5H), 5.90–5.85 (m, 1H), 5.74 (d, *J* = 5.3 Hz, 1H), 4.78–4.61 (m, 2H), 4.55–4.38 (m, 3H), 3.55 (t, *J* = 5.8 Hz, 2H), 3.23–3.14 (m, 1H), 2.47 (m, 1H), 2.22–2.10 (m, 3H), 1.83 (q, *J* = 6.1 Hz, 2H), 1.17 (dd, *J* = 6.9, 8.9 Hz, 6H). MS [M+H]^+^ 618.1.

#### H-phosphonate S3.

To a solution of **S2** (25 g, 0.040 mol, 1.0 equiv) in ethanol (500 mL) was added palladium on carbon (10%, 38 g) and acetic acid (25 mL, 26.3 g, 0.44 mol, 10.8 equiv). After stirring at 50°C under hydrogen for 2 d, the mixture was filtered, concentrated, and purified by silica gel column chromatography (petroleum ether/ethyl acetate, 5/1 to 2/1) to give the corresponding alcohol (20 g, 94% yield) as a white solid. MS [M+H]^+^ 528.3.

To a solution of the alcohol obtained above (3.0 g, 5.7 mmol, 1 equiv) in tetrahydrofuran (90 mL) was added imidazole (1.16 g, 17 mmol, 3.0 equiv) and triphenylphosphine (4.47 g, 17 mmol, 3 equiv) followed by a solution of iodine (2.6 g, 10 mmol, 1.8 equiv) in tetrahydrofuran (10 mL). After stirring at 25°C for 16 h, the reaction was quenched with saturated sodium sulfite (8 mL), concentrated, redissolved in ethyl acetate (80 mL), washed with water, dried over sodium sulfate, filtered, concentrated, and purified by silica gel column chromatography (petroleum ether/ethyl acetate, 1/1 to 1/3) to give the corresponding iodide (2.3 g, 63% yield) as a yellow solid. MS [M+H]^+^ 638.2.

To a solution of the iodide obtained above (3.8 g, 6.0 mmol, 1.0 equiv) in tetrahydrofuran (40 mL) was added sodium azide (2.5 g, 39 mmol, 6.5 equiv) in water (10 mL). After stirring at 50°C for 2 h, saturated sodium carbonate (50 mL) was added and the mixture was extracted with ethyl acetate (100 mL × 3), dried over sodium sulfate, filtered, and concentrated to give the crude azide (2.0 g, 61% yield) as a yellow solid. MS [M+H]^+^ 553.1.

To a solution of the azide obtained above (2.55 g, 4.6 mmol, 1.0 equiv) in ethanol (220 mL) was added sodium hydroxide (2 M, 23 mL, 46 mmol, 10 equiv) at 0°C. After stirring at 25°C for 30 min, the reaction was quenched with formic acid at 0°C, concentrated, and purified by preparative reverse-phase HPLC (0.1% trifluoroacetic acid in acetonitrile/water, 0 to 70%) to give the corresponding diol (1.6 g, 85% yield) as a white solid. MS [M+H]^+^ 407.1.

To a solution of the diol obtained above (1.6 g, 3.9 mmol, 1.0 equiv) in pyridine (15 mL) was added 4,4′-dimethoxytrityl chloride (1.6 g, 4.72 mmol, 1.2 equiv). After stirring at 25°C for 3 h, the reaction was quenched with methanol, concentrated, and purified by silica gel column chromatography (petroleum ether/ethyl acetate/ethanol, 1/1/0 to 0/250/1) to give the corresponding DMTr ether (0.95 g, 34% yield) as a yellow solid. MS [M+H]^+^ 709.4.

To a solution of the DMTr ether obtained above (700 mg, 0.99 mmol, 1 equiv) was added diphenyl phosphite (820 mg, 3.5 mmol, 3.5 equiv). After stirring at 25°C for 1.5 h, the mixture was concentrated, redissolved in methylene chloride (20 mL), washed with 5% sodium bicarbonate (20 mL), and concentrated. The resulting residue was redissolved in a mixture of dichloroacetic acid (0.24 mL), methylene chloride (10 mL), and water (3 mL). After stirring at 25°C for 30 min, the reaction was quenched with triethylamine (3 mL), concentrated, and purified by reverse-phase silica gel column chromatography (0.1% triethylamine in acetonitrile/water, 0 to 70%) to give **S3** (400 mg, 82% yield, ~95% pure) as a white solid. MS [M+H]^+^ 471.0.

#### Linear dinucleotide S4.

To a solution of **S3** (400 mg, 0.73 mmol, 1.0 equiv) in acetonitrile (3 mL) was added tetrazole (0.45 M in acetonitrile, 6.5 mL, 4.0 equiv). After stirring at 25°C for 5 min, *N*^6^-benzoyl-2′-*O*-*tert*-butyldimethylsilyl-5′-*O*-DMT-adenosine 3′-CE phosphoramidite (595 mg, 0.66 mmol, 0.9 equiv) was added. After stirring at 25°C for 30 min, *tert*-butyl hydroperoxide (0.21 mL, 2.18 mmol, 3 equiv) was added and the mixture was stirred at 25°C for 30 min before dichloroacetic acid (0.6 mL, 0.94 g, 7.3 mmol, 10 equiv) in methylene chloride (10 mL) was added. After stirring for 25 min, the reaction was quenched with saturated sodium sulfite (2 mL) and pyridine (2 mL), concentrated, and purified by reverse-phase silica gel chromatography (0.1% triethylamine in acetonitrile/water, 0 to 70%) to give **S4** (600 mg, 62% yield, ~80% pure) as a white solid. MS [M+H]^+^ 1071.5.

#### CDN S5.

To a solution of **S4** (400 mg, 0.37 mmol, 1.0 equiv) in pyridine (9 mL) was added 5,5-dimethyl-2-oxo-2-chloro-1,3,2-dioxaphosphinane (345 mg, 1.87 mmol, 5.0 equiv). After stirring at 25°C for 15 min, iodine (380 mg, 1.49 mmol, 4.0 equiv) and water (13.5 μL, 13.5 mg, 0.75 mmol, 2 equiv) were added and the mixture was stirred for 30 min before quenched with saturated sodium bicarbonate (2 mL) and sodium sulfite (2 mL), concentrated, and redissolved in acetonitrile (10 mL). tert-Butylamine (10 mL) was then added, and the mixture was stirred at 25°C for 1 h before concentrated and purified by reverse-phase silica gel column chromatography (0.1% triethylamine in acetonitrile/water, 0 to 70%) to give **S5** (350 mg, 83% yield, ~90% pure) as a white solid. MS [M+H]^+^ 1016.4.

#### IMSA172.

To **S5** (300 mg, 0.3 mmol, 1.0 equiv) was added a solution of methylamine in ethanol (5 M, 3 mL, 15 mmol, 50 equiv). After stirring at 25°C for 2 h, the mixture was concentrated and purified by reverse-phase silica gel column chromatography (0.1% triethylamine in acetonitrile/water, 0 to 35%) to give the TBS-protected azido-**IMSA172** (160 mg, 52% yield, ~80% pure) as a white solid. MS [M+H]^+^ 842.3.

To a solution of TBS-azido-**IMSA172** (100 mg, 0.12 mmol, 1.0 equiv) in methanol (8 mL) was added palladium on carbon (10%, 30 mg). After stirring at 25°C under hydrogen for 5 h, the mixture was filtered, concentrated, and purified by reverse-phase silica gel column chromatography (0.1% triethylamine in acetonitrile/water, 0 to 30%) to give TBS-**IMSA172** (70 mg, 65% yield, ~90% pure) as a white solid. MS [M+H]^+^ 816.5.

To a solution of TBS-**IMSA172** (35 mg, 0.043 mmol, 1.0 equiv) in methanol (3 mL) was added ammonium fluoride (127 mg, 3.4 mmol, 80 equiv). After stirring at 70°C for 15 min, the mixture was concentrated and purified by reverse-phase silica gel column chromatography (0.1% formic acid in acetonitrile/water, 0 to 30%) to give **IMSA172** (8.6 mg, 28% yield) as a white solid. MS [M+H]^+^ 702.0. ^1^H NMR (400M, D_2_O + K_2_HPO_4_ buffer): δ 8.26 (s, 2H), 7.85 (s, 1H), 6.16 (s, 1H), 5.95–5.85 (m, 1H), 5.80–5.70 (m, 1H), 5.13–5.05 (m, 1H), 4.53–4.40 (m, 2H), 4.40–4.35 (m, 1H), 4.30–4.20 (m, 1H), 4.16–4.07 (m, 2H), 3.25–3.15 (m, 2H), 2.85–2.70 (m, 1H), 2.45–2.32 (m, 1H), 1.97–1.85 (m, 1H). ^31^P NMR (162 MHz, D_2_O + K_2_HPO_4_ buffer): δ–1.04, –2.36 (*SI Appendix*, Fig. S2).

### Synthesis of IMSA-172-Linker.

The reaction scheme for the synthesis of IMSA172-linker is shown in *SI Appendix*, Fig. S3. Detailed synthetic procedures are described below.

#### Mc-Val-Cit-PABC-IMSA172.

A solution of **IMSA172** (90 mg, 0.13 mmol, 1.0 equiv), Mc-Val-Cit-PABC-PNP (189 mg, 0.26 mmol, 2.0 equiv), hydroxybenzotriazole (17.3 mg, 0.13 mmol, 1.0 equiv), and *N*,*N*-diisopropylethylamine (0.22 mL, 1.28 mmol, 10.0 equiv) was stirred under nitrogen for 42 h before diluted with a mixture of ethyl acetate (75 mL), *tert*-butyl methyl ether (225 mL), and acetic acid (0.1 mL). The solid was collected, washed with *tert*-butyl methyl ether (80 mL × 2), extracted twice with a mixture of methanol (2 mL), and water (10 mL) under sonication, concentrated, and purified by ion-exchange chromatography on a Mono Q column (sodium chloride/20 mM pH 7.4 HEPES buffer, 0 to 0.5 M) to give Mc-Val-Cit-PABC-IMSA172 as a solution containing 0.24 M sodium chloride. MS [M+H]^+^ 1300.4.

### Cell Lines.

B16F10 melanoma cell line was purchased from ATCC. B16-EGFR and MC38 EGFR cells were gifts from Dr. Yang-Xin Fu. These cells were cultured in DMEM containing 10% Fetal Bovine Serum (FBS, Sigma) and antibiotics. THP1-ISG-luc and Raw-ISG-luc reporter cells were purchased from Invivogen (San Diego, CA) and cultured in RPMI with 5% FBS and antibiotics. THP1-ISG-luc-EGFR cell line was constructed by transduction of human EGFR into THP1-ISG-Luc cells using the lentiviral system described previously (31).

### Reporter Assays for STING Activation in Cells.

Serial dilutions of ADCs, antibodies, or small molecules were added to 0.3 **×** 10^6^ cells in 0.1 mL of media and incubated for 16 h. In some cases, 25 ng/mL PFO was used to permeabilize cells and facilitate the entry of small molecules. 20 μL of media was mixed with 100 μL of 1 μM native coelenterazine (Gold Biotechnology, St Louis MO) in PBS as substrate and luminescence was measured immediately.

### Expression and Purification of Antibodies.

Mouse anti-EGFR was purchased from BioXcell (Cat# BE-0279). Amino acid sequences of the heavy chain and light chain of hu-anti-EGFR (cetuximab) were retrieved from a database (32), cDNAs encoding both original cetuximab and the ACVC variant (A114C,V205C) were synthesized and cloned into the vector pGenHT1.0-DGV. Plasmids were transfected into CHO-K1SP cells to establish a stable cell line with the selection drug L-Methionine sulfoximine. To produce antibodies, stable cells with a starting density of 0.5 **×** 10^6^ were cultured in Dynamis™ medium (Gibco 26615-01) for 14 d. EfficientFeed™ B media supplemented with AGT™ (Gibco A25030-05) was added on day 3 (3% of initial volume), day 5 (4%), day 7 (5%), and day 9 (5%). The final culture supernatant was loaded onto a 5 mL protein A column (Cytiva); after washing with 10 column volumes of PBS, the antibody was eluted with 0.1 M Glycine (pH2.5) and further purified using a 5mL Hitrap® desalting column (Cytiva).

### Preparation of STING ADCs.

#### Random conjugation.

The reaction scheme is shown in *SI Appendix*, Fig. S3. The anti-EGFR antibodies were reduced with four molar equivalents of tris-(2-carboxyethyl)-phosphine hydrochloride (TCEP) at 37°C for 2 h. IMSA172-linker was added at 4.2 molar equivalent and incubated at room temperature for 1 h. Small molecules were removed by a desalting column in PBS. ADC was further purified by loading onto a 1 mL protein A column [Cytiva equilibrated with PBS, and sequentially washed with 15 mL of wash buffer (PBS with 0.35M NaCl and 0.2% Triton X-100] and 15 mL of PBS, followed by elution with 5 mL of 0.1 M Glycine, pH2.5. The eluted ADC was subjected to size exclusion chromatography using a Superdex 200 column (Cytiva). A single peak at 12.3 mL was collected as purified ADC.

#### Site-specific conjugation.

Human anti-EGFR (ACVC) was reduced with 50 molar equivalents of TCEP at 37°C for 2 h. Excess TCEP was removed using a desalting column in conjugation buffer [50 mM Hepes-NaOH, pH7.4, 50 mM NaCl]. The recovered antibody was re-oxidized with 50 molar equivalents of dehydroascorbic acid (DHA) at room temperature overnight, followed by incubation with 4.2 molar equivalent of IMSA172-linker at 37°C for 2 h. After the reaction was quenched with 100 molar equivalents of N-acetyl cysteine (NAC), conjugated ADC was further purified as described above.

#### DAR estimation.

DAR of ADC was calculated by comparing the OD260/280 ratio with a standard curve generated by mixing antibody and IMSA172 linker at known ratios.

### Mice.

C57BL/6J mice were purchased from The Jackson Laboratory (Bar Harbor, ME) and maintained in the animal care facility of the University of Texas Southwestern Medical Center at Dallas. Experimental protocols were approved by the Institutional Animal Care and Use Committee.

### Antitumor Efficacy Experiments in Syngeneic Mouse Tumor Models.

B16F10 or B16-EGFR cells were grown in DMEM containing 10% FBS. 10^6^ of log-phase tumor cells in 100 μL of PBS were injected subcutaneously into C57BL6 mice at their right flanks. 6–7 d after tumor inoculation, mice were re-grouped with matched tumor volumes. ADC or PD-L1 antibodies were each diluted to 2 mg/mL in PBS and administered to mice intraperitoneally (I.P.) at 200 μg per mouse. In some control groups, Antibody and IMSA172 were mixed in 100 μL PBS and injected intraperitoneally. Tumors were measured every 2–3 d with a digital caliper (Fisher Scientific), and the tumor sizes were calculated using the following formula: π/6 × length × width × height. Mouse survival was monitored daily.

### PKs/PDs of ADC in Mice.

10^6^ B16-EGFR cells in 100 μl PBS were injected into the right flank of a mouse subcutaneously. Six days after inoculation, mice with established tumors were injected intraperitoneally with 100 μg of the hu-anti-EGFR(ACVC)-172 ADC. At each indicated time point, blood samples were collected in the presence of heparin as the anti-coagulant and spun at 5000 **×** g for 5 min to obtain plasma. Tumors were harvested from mice after they were sacrificed at indicated time points. Tumors were homogenized in a buffer [50 mM Tris-Cl, pH7.5, 100 mM NaCl, 0.5% Triton X100, 0.1 mM DTT, and protease inhibitor cocktail] and spun at 12,000 g for 5 min to obtain cell lysates. Plasma and tumor lysates were subjected to enzyme-linked immunosorbent assay (ELISA) to measure concentrations of ADC and cytokines. Briefly, 96-well plates were coated with EGFR extracellular domain (ECD, Sino Biological, Wayne, PA), 100 ng/well in 100 μL of PBS overnight. After blocking with 3% BSA in PBS for 1 h, plates were washed with PBS containing 0.05% Tween-20 and incubated with diluted plasma, tumor lysates, or ADC standards for 1.5 h at room temperature. Plates were again washed and incubated with anti-human-IgG labeled with HRP (ThermoFisher, Waltham, MA). After final washes, 100 μL of the substrate TMB (3,3′,5,5′-Tetramethylbenzidine, ThermoFisher) was added and incubated for 15 min, followed by the addition of 100 μL of 1.0 M H_2_SO_4_, and OD450 was measured. IFNβ and IL-6 were measured using ELISA kits purchased from Invivogen and BD Biosciences, respectively, following manufacturer’s instructions. For quantification of the ADC activity, diluted plasma samples were incubated with THP1-ISG-luc-EGFR cells for 16 h. Luciferase activity in media was measured and concentrations of active ADC were extrapolated using the standard curve of hu-anti-EGFR(ACVC)-172 ADC, which was tested through serial dilutions in the same assay. To measure free payload in plasma, samples were filtered through a 10kDa cutoff membrane cassette (Amicon®) and incubated with THP1-ISG-luc cells in the presence of PFO for 16 h followed by measurement of luciferase activity in the media.

### Immune Cell Profiling Using Flow Cytometry.

For tumor-infiltrating immune cell analyses, tumors were cut into 3–4-mm pieces and digested with 200 U/mL collagenase IV (in HBSS with 1% FBS) for 30 min at 37°C. Digested tumors were minced and filtered through 100-μm cell strainers (Greiner Bio-One) to obtain single-cell suspensions. Draining lymph nodes were minced and filtered through 40-μm cell strainers to get single-cell suspensions. All the following staining steps were done in MACS buffer (PBS + 2% FBS+ 1 mM EDTA). Cells were stained with zombie yellow (BioLegend, cat# 423103) to label dead cells. After washing with MACS buffer, the Fc receptors on the cell surface were blocked with anti-CD16/32 antibody (10 µg/mL) and then stained with fluorophore-labeled antibodies listed in Table 1. When more than two BUV labeled antibodies were used in the same experiment, 10**×** Brilliant Stain Buffer Plus (BD biosciences, cat# 566385) were added to the antibody mixture to avoid reactions among BUV reagents. Data were acquired with a Cytek Aurora (5-laser) flow cytometer and analyzed with FlowJo software.

**Table 1.** Antibodies against cell surface proteins used in FACS analyses

CD marker	Fluorophore	Vendor	Catalog No.
CD45	BV785	Biolegend	109839
F4/80	BV421	BD Biosciences	565411
CD11b	Percp	Biolegend	101230
CD11c	Percp-cy5.5	Biolegend	117327
IA/IE	FITC	Biolegend	107606
CD206	PE	Biolegend	141705
CD206	Spark YG™ 570	Biolegend	141737
NK1.1	Alexa Fluor 647	Biolegend	108720
NK1.1	BUV615	BD Biosciences	751111
CD69	Pacific blue	Biolegend	104524
CD8	Alexa Fluor 700	Biolegend	100730
CD19	BV750	Biolegend	115561
CD3e	BUV496	BD Biosciences	612955
CD4	APC/Fire™ 810	Biolegend	100479
PD-L1	BUV395	BD Biosciences	745616
CD80	BV711	Biolegend	104743
CD86	BUV805	BD Biosciences	749021

## Supplementary Material

Appendix 01 (PDF)

## Data Availability

All study data are included in the article and/or *SI Appendix*.
