# Supplementary material for: Tumor-targeted delivery of a STING agonist improves cancer immunotherapy
Source: Proc Natl Acad Sci U S A. 2022 Nov 29;119(49):e2214278119. doi: 10.1073/pnas.2214278119 (PMC9894229; doi:10.1073/pnas.2214278119)
Supplement: Supplementary file 1 — Appendix 01 (PDF) [file pnas.2214278119.sapp.pdf]

## SUPPLEMENTARY FIGURES

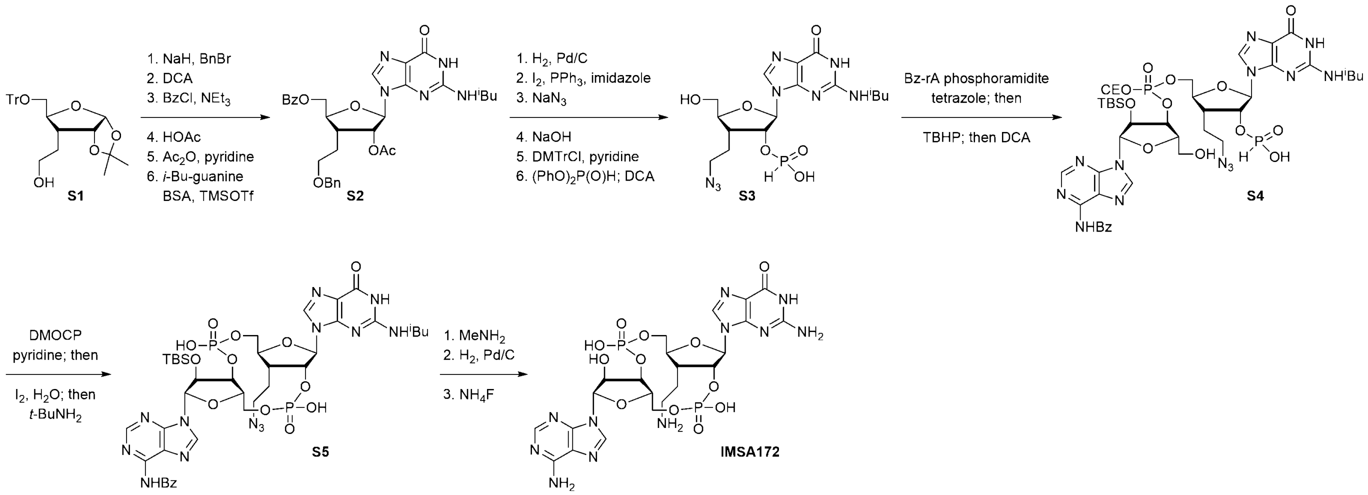


**Figure S1. Reaction scheme for the synthesis of IMSA172**. The detailed procedures are described in Materials and Methods.

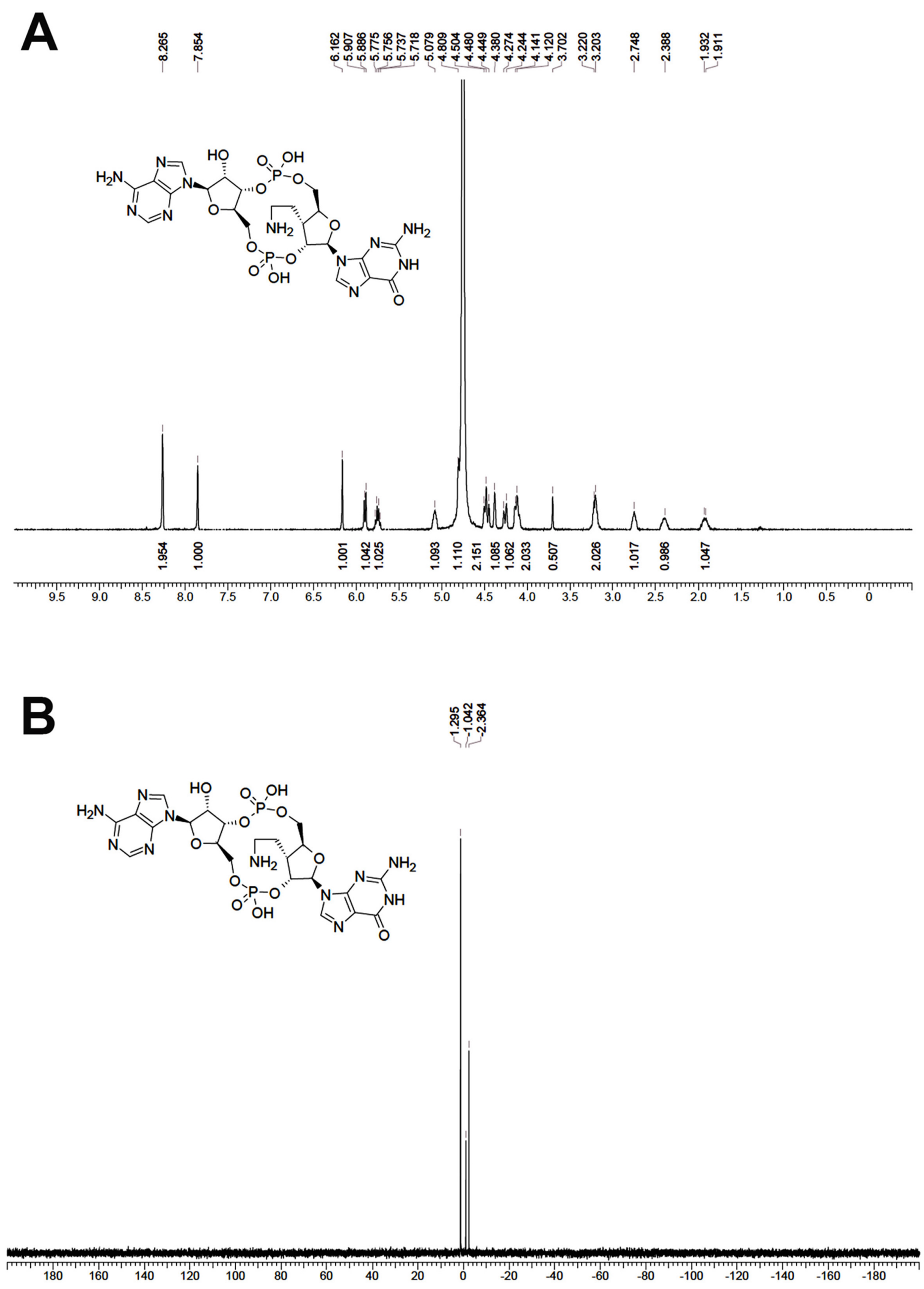


**Figure S2. NMR spectra of chemically synthesized IMSA172**. (A) $^{1}$H NMR spectrum of IMSA172. Chemical shifts are reported in ppm (δ) referenced to the residual signal of the solvent ($D_2O$). $^{1}$H NMR ($D_2O$ + $K_2HPO_4$ buffer, 400MHz): δ (ppm) 8.26 (s, 2H), 7.85 (s, $^{1}$H), 6.16 (s, $^{1}$H), 5.85-5.95 (m, $^{1}$H), 5.70-5.80 (m, $^{1}$H), 5.05-5.13 (m, $^{1}$H), 4.40-4.53 (m, $^{2}$H) , 4.35-4.40 (m, $^{1}$H), 4.20-4.30 (m, $^{1}$H), 4.07-4.16 (m, $^{2}$H), 3.15-3.25 (m, $^{2}$H), 2.70-2.85 (m, $^{1}$H), 2.32-2.45 (m, $^{1}$H), 1.85-1.97 (m, $^{1}$H). (B) $^{31}$P NMR spectrum of IMSA172. Chemical shifts are reported in ppm (δ) referenced to the $K_2HPO_4$ buffer. $^{31}$P NMR ($D_2O$ + $K_2HPO_4$ buffer, 400MHz): δ (ppm) -1.04, -2.36.

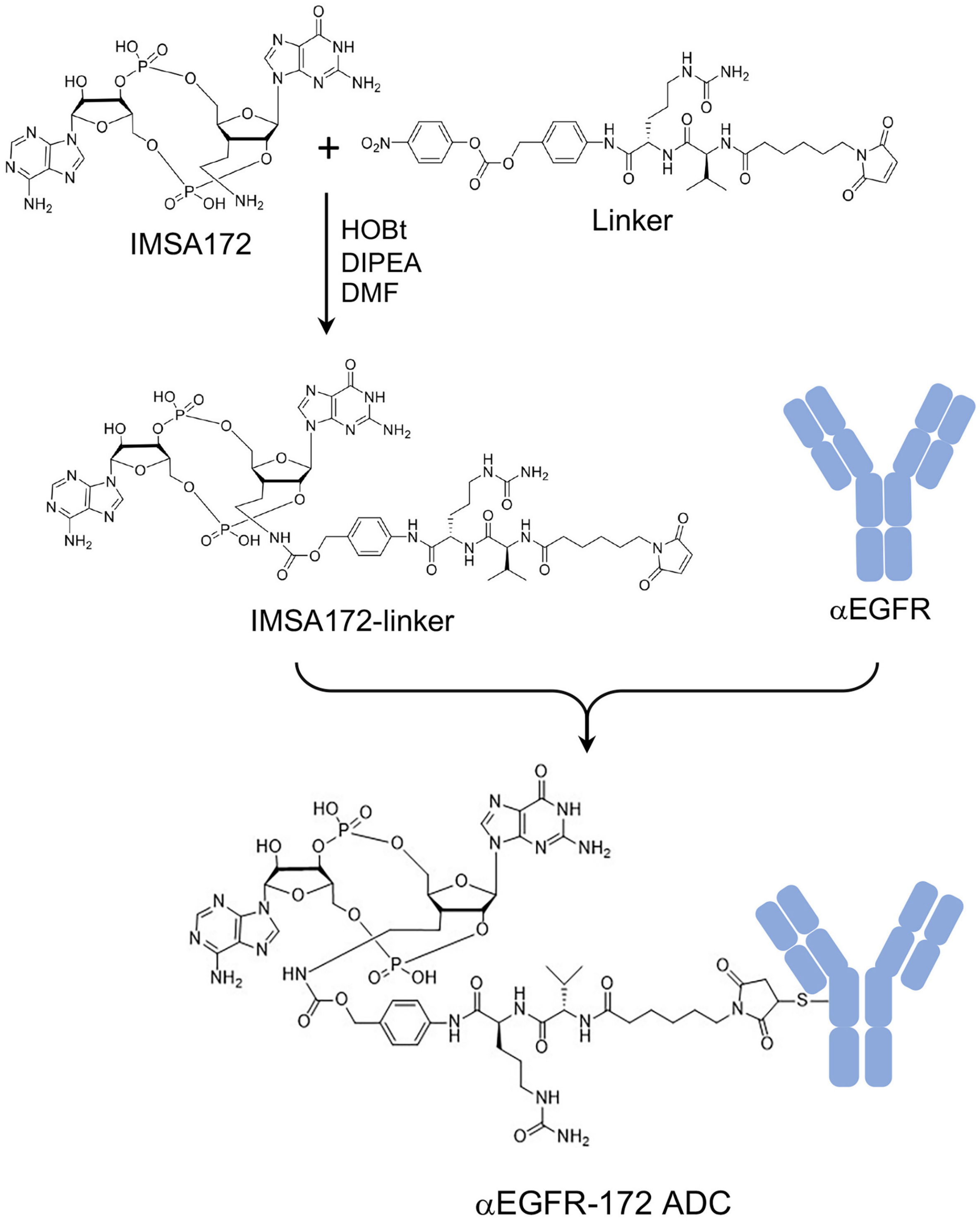


**Figure S3. Reaction schemes for the synthesis of IMSA172-linker and ADC**. The detailed protocols are described in Materials and Methods.

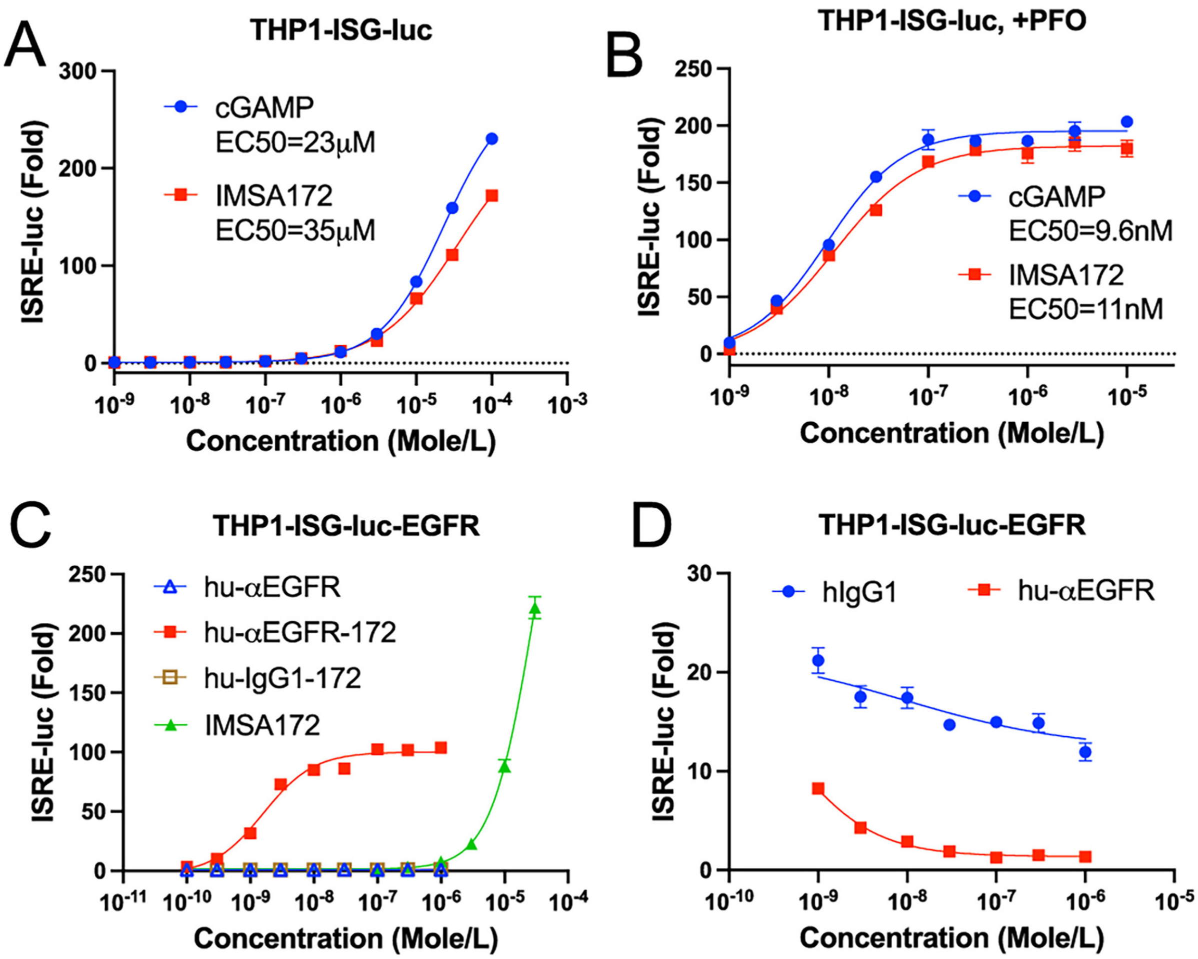


**Figure S4**. EGFR-172 ADC potently activates the STING pathway in EGFR expressing cells. (A) Indicated concentrations of cGAMP or IMSA172 were incubated with the THP1-ISG-luc cells for 16 hours, followed by measurement of luciferase activity in the media. (B) Similar to (A) except that 25ng/mL of perfringolysin O (PFO) was used to permeabilize the THP1 reporter cells to facilitate the compound entry to the cells. (C) Indicated concentrations of hu-αEGFR, hu-αEGFR-172 (ADC), hu-IgG1-172 (control ADC) or IMSA172 were incubated with the THP1-ISG-luc-EGFR cells for 16 hours, followed by measurement of luciferase activity in the media. (D) Indicated concentrations of the hu-αEGFR antibody or IgG1 antibody (as a control) was incubated with THP1-ISG-luc-EGFR cells for 30 minutes before 1.0 nM of hu-αEGFR-172 was added to the cell culture. 16 hours later, luciferase activity in the media was measured. Data are shown as mean ± SEM in triplicate assays. ns: not significant, *P<0.05, **p<0.01, ***p<0.001, ****p<0.0001 (student's *t* test).

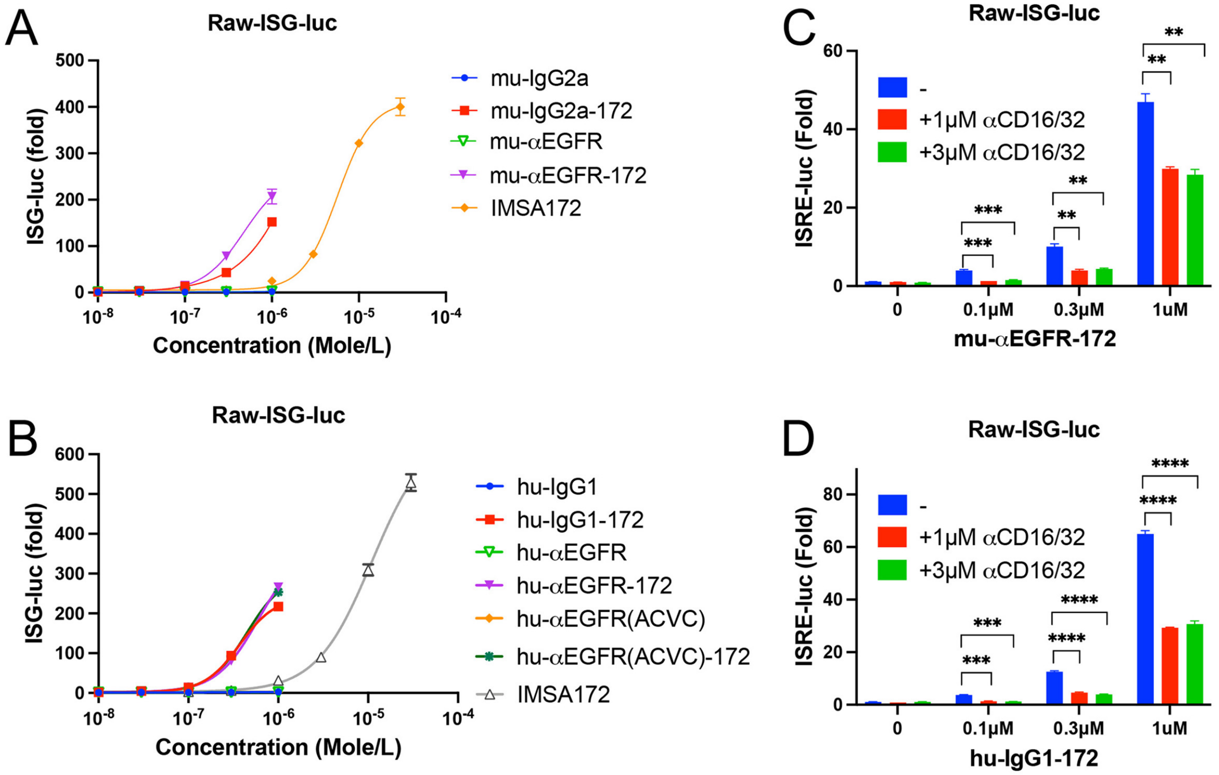


**Figure S5**. STING ADC can activate macrophages through the Fc receptors at high concentrations. (A) Indicated concentrations of mu-αEGFR-172 (ADC), mu-IgG2a-172 (control ADC), unconjugated antibodies or the free payload (IMSA172) were incubated with Raw-ISG-luc cells for 16 hours, followed by measurement of luciferase activity. (B) Similar to (A), except that different ADCs and unconjugated antibodies were used as indicated. (C and D) The Fc receptor blocking antibodies, αCD16/32, were incubated with Raw-ISG-luc cells at the indicated concentrations for 30 minutes before different amounts of mu-αEGFR-172 (C) or hu-IgG1-172 (D) were added to the cell culture. 16 hours later, luciferase activity in the media was measured. Data are shown as mean ± SEM in triplicate assays. ns: not significant, *P<0.05, **p<0.01, ***p<0.001, ****p<0.0001 (student's *t* test).

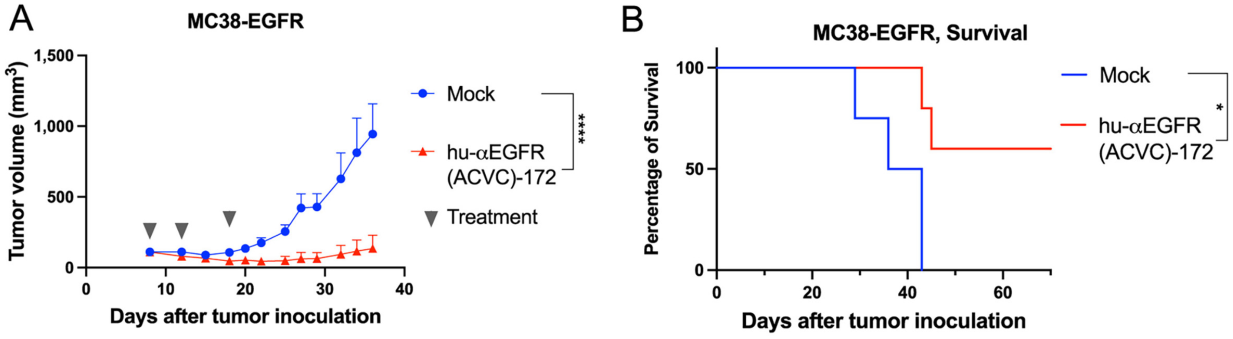


**Figure S6**. αEGFR-172 ADC inhibits the growth of MC38 tumors expressing EGFR. C57BL/6 mice (n=5) were inoculated subcutaneously with MC38 tumor cells stably expressing human EGFR. After the tumors grew to ~110 $mm^3$, the mice were injected intraperitoneally with PBS (Mock) or hu-αEGFR(ACVC)-172; Tumor growth (A) and mouse survival (B) were monitored. Tumor growth data are presented as mean ± SEM, ****$p < 0.0001$ (two-way ANOVA). Log rank (Mantel-Cox) test was used for survival data, ns: not significant, *$p<0.0332$.

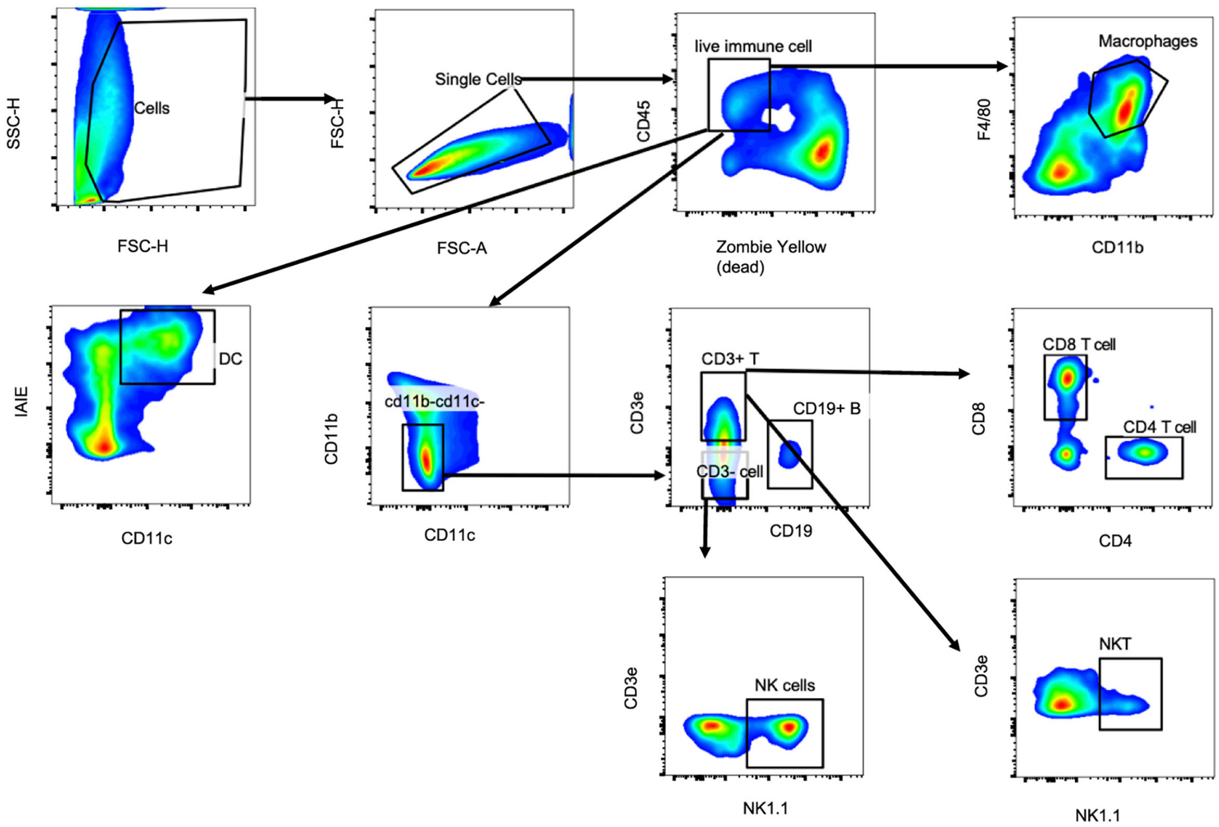


**Figure S7. Gating strategy for analyzing immune cell population in tumors and draining lymph nodes.** T cells, NK cells, NKT cells, dendritic cells, and macrophages were gated based on markers shown in the plot.

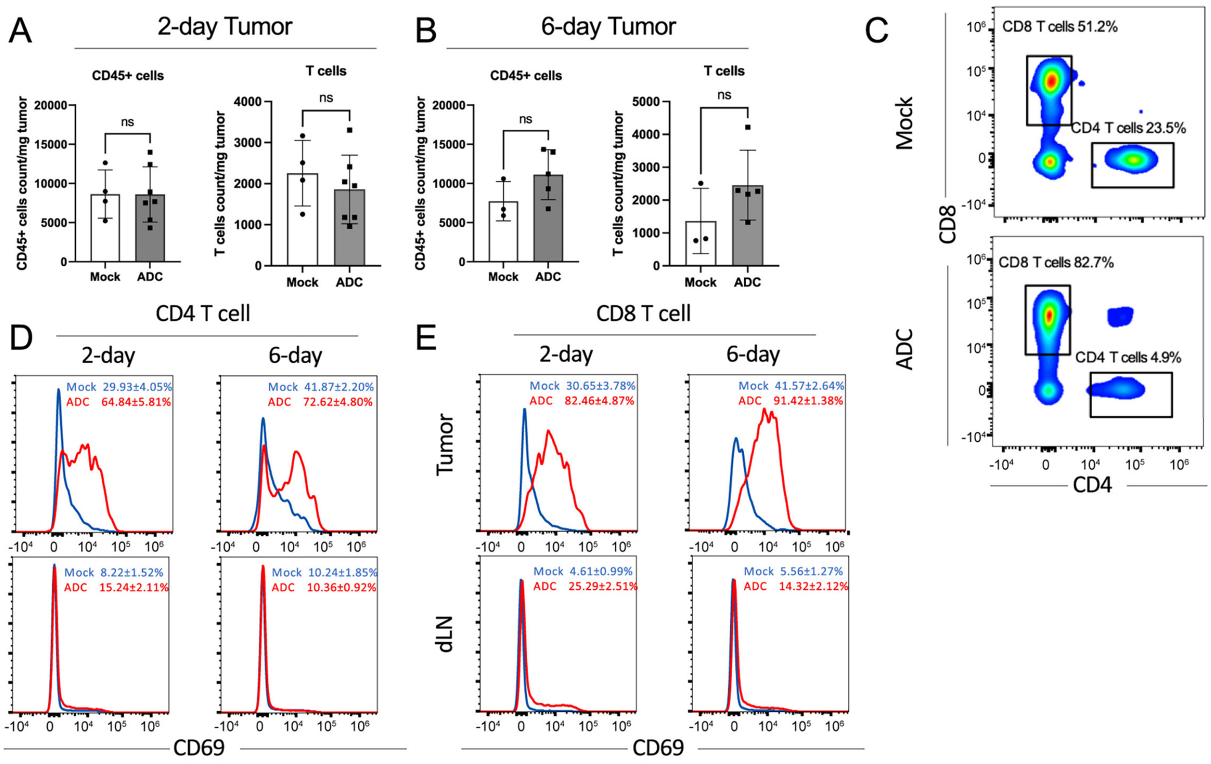


**Figure S8. αEGFR-172 ADC activated T cells and increased CD8/CD4 ratio in tumors.** Total immune cell (CD45+) counts, and T cells (CD3+) counts per mg tumor tissue at 2 days (A) or 6 days (B) after the first ADC or mock treatment. Data are shown as mean ± SD and individual values. ns: not significant (student's *t* test). (C) Representative FACS plots of CD4 and CD8 T cells under the CD45+CD11b-CD11c-CD3+CD19- gate. (D and E) Representative histograms of CD69 expression levels on CD4 T cells (D) and CD8 T cells (E) from tumors and draining lymph nodes (dLNs). The x-axis shows the fluorescence intensity of the anti-CD69 antibody. The y-axis shows the normalized cell counts. The percentage of CD69+ cells are shown as mean ± SD in each plot. 2-day mock: n=4, 2-day ADC: n= 7, 6-day mock: n=3, 6-day ADC: n= 5.

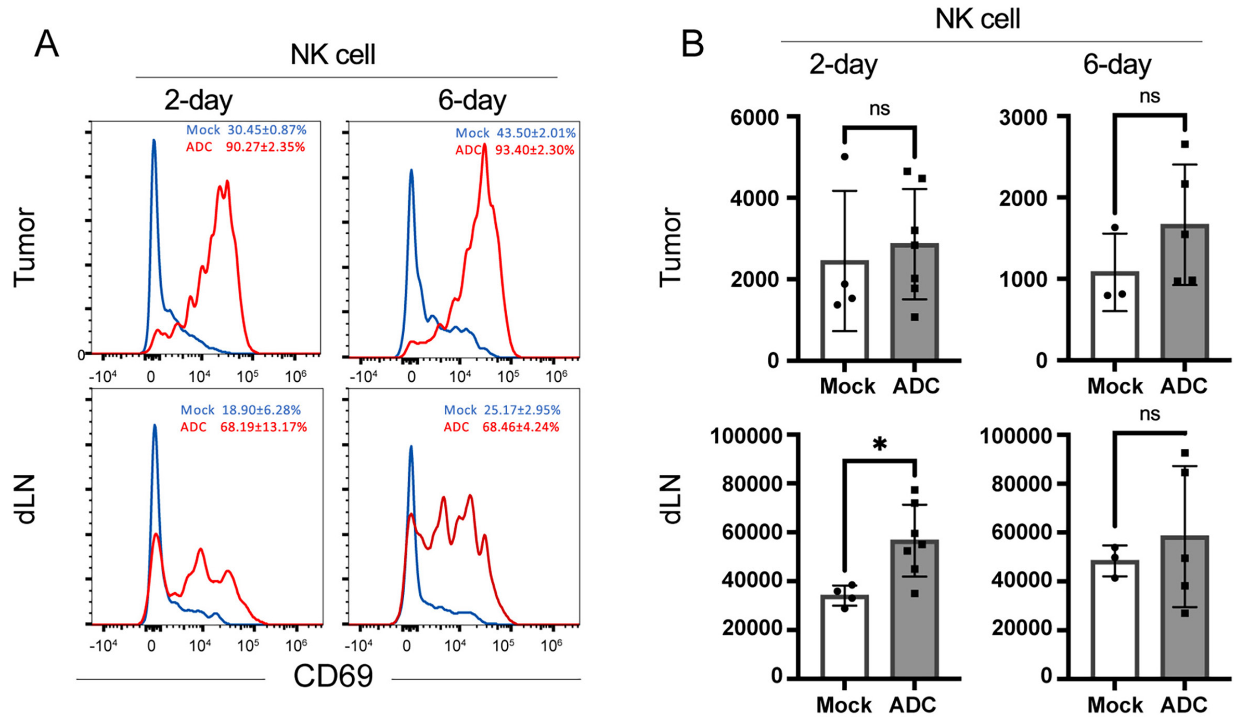


**Figure S9. αEGFR-172 ADC activated NK cells in tumors and draining lymph nodes. (A)** Representative histograms of CD69 expression levels in NK cells. The x-axis represents the fluorescence intensity of the anti-CD69 antibody, and the y-axis shows the normalized cell counts. The percentage of CD69+ cells are shown as mean ± SD in each plot. (B) Total NK cell (NK1.1+CD3-) counts per mg tumor tissue or per dLN at 2 days or 6 days after the first ADC or mock treatment. Data are shown as mean ± SD and individual values. 2-day mock: n=4, 2-day ADC: n= 7, 6-day mock: n=3, 6-day ADC: n= 5.  ns: not significant, *P<0.05 (student's *t* test).

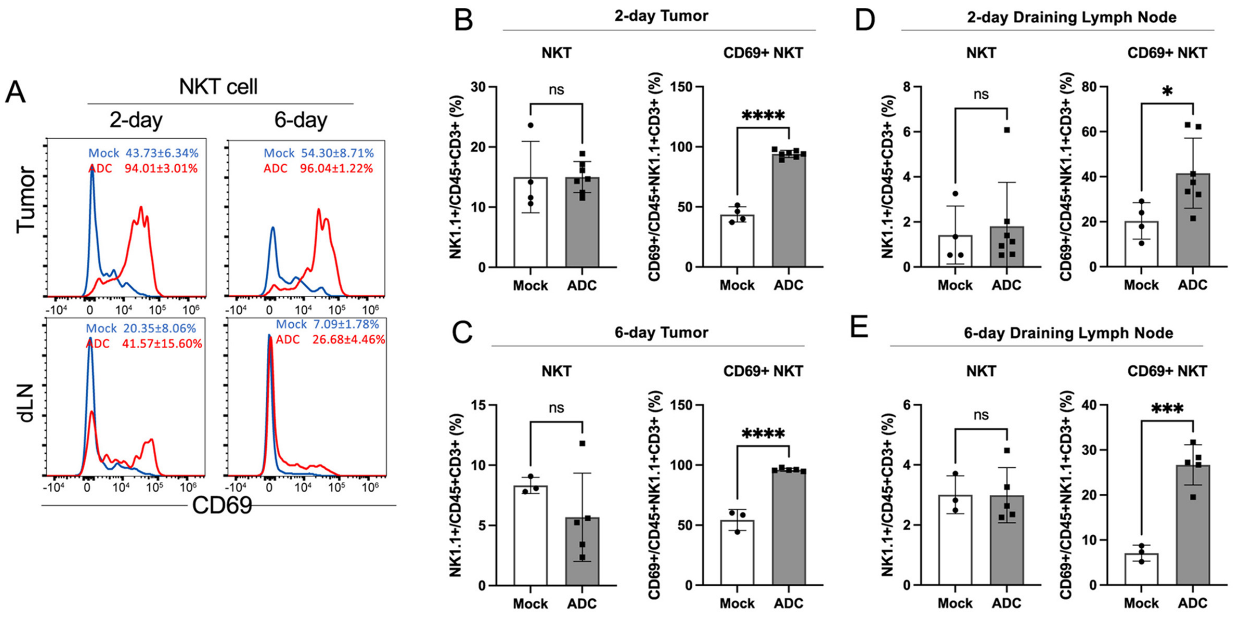


**Figure S10. αEGFR-172 activated NKT cells in tumors and draining lymph nodes.** (A) Representative histograms of CD69 expression levels in NKT cells. The percentage of CD69+ cells are shown as mean ± SD in each plot. (B to E) The percentages of NKT cells in T cells and activated NKT cells in NKT cells after 2 days (B and D) or 6 days (C and E) of ADC or mock treatment in tumors (B and C) and draining lymph nodes (dLNs; D and E). 2-day mock: n=4, 2-day ADC: n= 7, 6-day mock: n=3, 6-day ADC: n= 5. Data are shown as mean ± SD and individual values. ns: not significant, *P<0.05, **p<0.01, ***p<0.001, ****p< 0.0001 (student's *t* test).

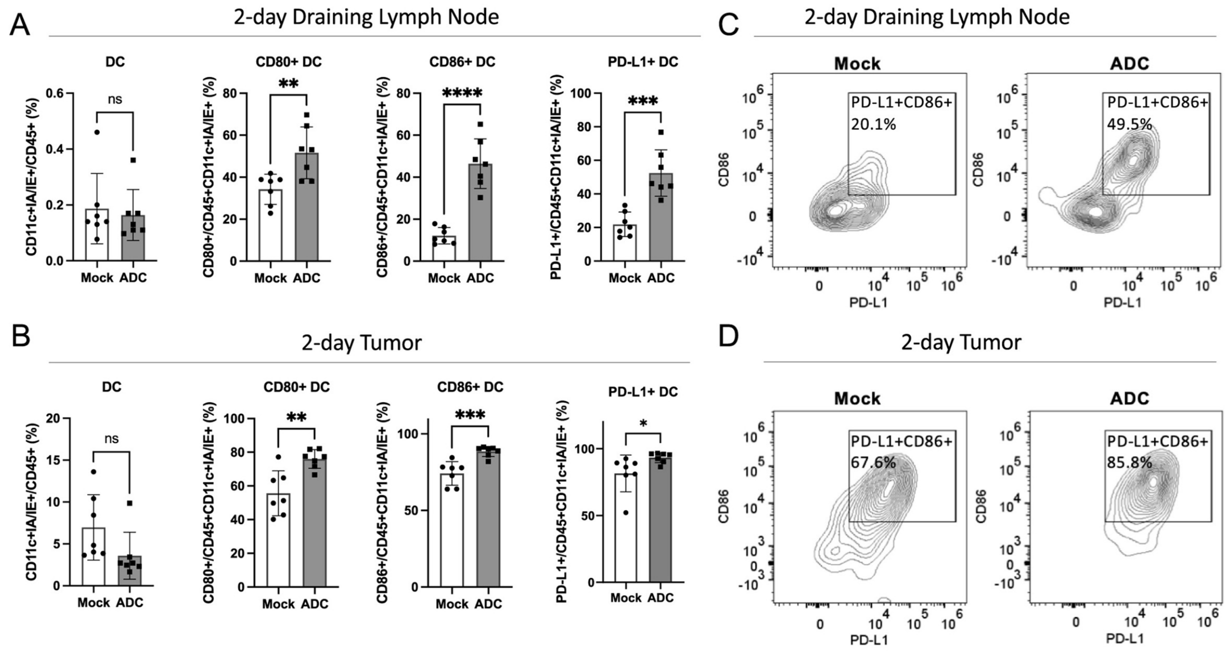


**Figure S11. αEGFR-172 ADC activated dendritic cells in tumors and draining lymph nodes.** (A-B) C57BL/6 mice bearing B16F10-EGFR tumors were treated with hu-αEGFR(ACVC)-172 (ADC) or PBS (mock) intraperitoneally. Two days after treatment, cells from the tumors (B) and draining lymph nodes (A) were analyzed by flow cytometry, focusing on dendritic cells (DCs, CD11c+IA/IE+) and their activation markers as indicated. Mock: n=7, ADC: n= 7. Data are shown as mean ± SD and individual values. ns: not significant, *P<0.05, **p<0.01, ***p<0.001, ****p< 0.0001 (student's *t* test). (C-D) Representative FACS plots of CD86 and PD-L1 expression levels in DCs from draining lymph nodes (C) and tumors (D).

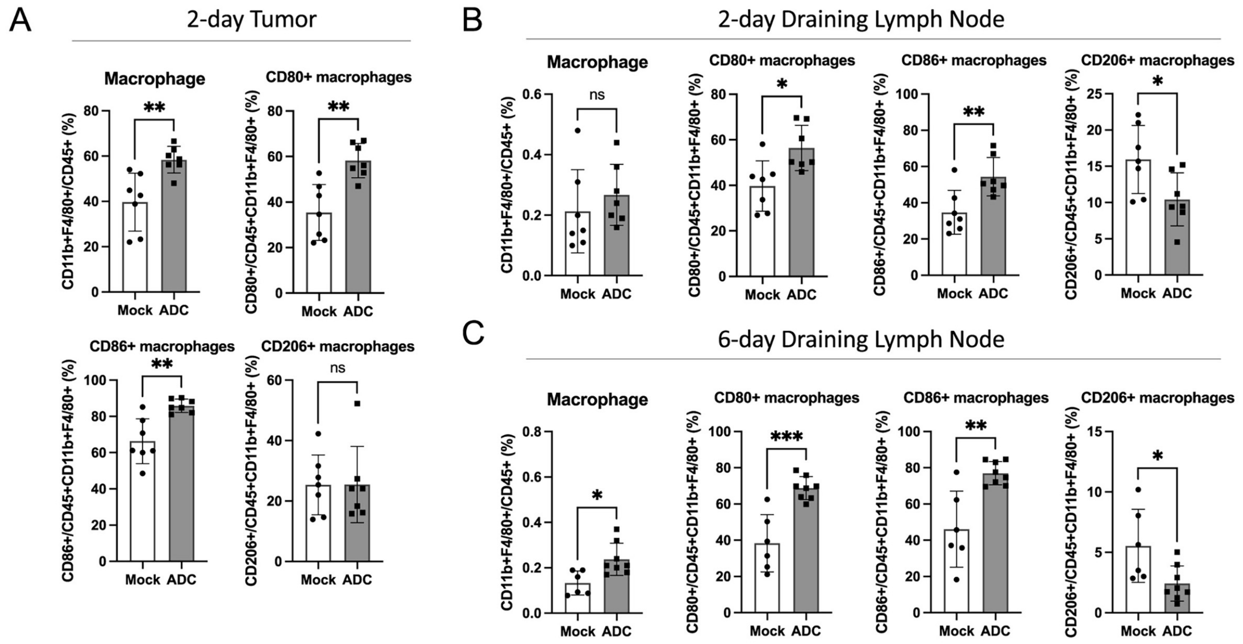


**Figure S12. αEGFR-172 ADC promoted the M2 to M1 polarization of macrophages in tumors and draining lymph nodes.** Experiments were performed as described in Figure S11, except that the flow cytometry analysis focused on macrophages (CD11b+F4/80+) and the markers for M1 (CD80 and CD86) and M2 (CD206) macrophages. 2-day mock: n=7, 2-day ADC: n= 7, 6-day mock: n=6, 6-day ADC: n= 8. Data are shown as mean ± SD and individual values. ns: not significant, *P<0.05, **p<0.01, ***p<0.001, ****p< 0.0001 (student's *t* test).
